# The human claustrum supports cognitive networks for externally and internally driven task demands

**DOI:** 10.1371/journal.pbio.3003843

**Published:** 2026-06-26

**Authors:** Brent W. Stewart, Matthew A. Cormie, Michael L. Keaser, Massieh Moayedi, Brian N. Mathur, David A. Seminowicz

**Affiliations:** 1 Department of Neural and Pain Sciences, University of Maryland School of Dentistry, Baltimore, Maryland, United States of America; 2 Centre for Multimodal Sensorimotor and Pain Research, Faculty of Dentistry, University of Toronto, Toronto, Ontario, Canada; 3 University of Toronto Centre for the Study of Pain, Toronto, Ontario, Canada; 4 Division of Clinical & Computational Neuroscience, Krembil Brain Institute, University Health Network, Toronto, Ontario, Canada; 5 Department of Pharmacology, University of Maryland School of Medicine, Baltimore, Maryland, United States of America; 6 Department of Psychiatry, University of Maryland School of Medicine, Baltimore, Maryland, United States of America; 7 Department of Medical Biophysics, Schulich School of Medicine & Dentistry, University of Western Ontario, London, Ontario, Canada; Oxford University, UNITED KINGDOM OF GREAT BRITAIN AND NORTHERN IRELAND

## Abstract

Cognitive control is believed to arise from task-dependent interactions among networks of brain regions. Although several debilitating neuropsychiatric disorders are characterized by cognitive network dysfunction, the neural circuit mechanisms supporting task-dependent network activity are largely unknown. External and internal task demands elicit opposing responses from key cognitive networks, and claustrum projections target regions associated with both network states. We tested if claustrum supports task-dependent network activity in humans using fMRI during tasks with externally and internally driven demands: working memory (*n* = 420) and autobiographical memory (*n* = 35). Claustrum activity increased in both tasks. Claustrum exhibited anatomical connectivity with regions representing all implicated networks, and claustrum effective connectivity suggested an excitatory influence on regions in multiple task-associated networks. Task response and connectivity measures differed between the claustrum and regions prominently implicated in directing network states—the anterior insula and pulvinar. These findings establish a role for the claustrum in supporting task-dependent network states subserving cognitive control.

## Abstract 2

Cognitive control depends on dynamic interactions among large-scale brain networks, but the circuit mechanisms enabling task-dependent network coordination in humans remain unclear. This study shows that claustrum activity increases in memory tasks and that it has an excitatory influence on regions in multiple task-associated networks.

## Significance

Cognitive function is supported by large-scale networks of regions across the brain. Yet, the neural circuit mechanisms supporting task-dependent network activity are largely unknown. Analyses of human functional and structural neuroimaging in this study found that the claustrum, a subcortical nucleus, responds to externally and internally driven cognitive task demands representing a wide range of network states, possesses strong anatomical connections with cognitive control network nodes, and exerts excitatory influence on task-associated regions in multiple networks. These results establish the claustrum as a contributor to task-dependent network activity subserving cognitive control.

## Introduction

Cognitive control is the brain’s ability to use internal goals to filter sensory inputs and select appropriate actions [1]. This ability is important to most aspects of human life [2], as evidenced by the variety of disorders associated with cognitive control dysfunction, such as schizophrenia [3], substance use disorders [4,5], depression [6], attention deficit hyperactivity disorder [7], obsessive-compulsive disorder [8], and chronic pain [9,10].

The human brain consists of large-scale networks of regions [11,12] differentially active depending on task demands [13]. Cognitive control is a higher-order function that requires greater coordination among brain-wide networks than simple motor tasks [14]. Therefore, a prominent hypothesis contends cognitive control arises from dynamic activation of multiple large-scale brain networks to meet task demands [15]. However, how the brain achieves task-dependent network states is not fully understood.

The claustrum is a global network hub, exhibiting the most extensive structural connectivity of all brain regions by volume [16] and resting state functional connectivity with multiple networks [17,18]. Synaptic connectivity between the claustrum and the cortex aligns with network motifs, and the claustrum is required for cognitively demanding task performance [17,19–25]. To test if the claustrum supports task-dependent network activity required for cognitive control [26], we analyzed task-dependent claustrum activity and connectivity in human fMRI data from two tasks evoking distinct network states and corresponding diffusion MRI (dMRI) scans.

A working memory task increased activity in regions associated with external attention [27], and an autobiographical memory task increased activity in regions associated with internally-directed thought [28]. The claustrum was active in both tasks and exhibited significant anatomical connections with regions representing all associated cognitive networks. Effective connectivity analyses suggested the claustrum exerted an excitatory influence on regions in multiple task-associated networks in both tasks. These findings support the hypothesis that the claustrum contributes to task-dependent network states that ultimately underpin cognitive control.

## Results

### Claustrum co-activates with externally driven working memory networks

**Fig 1:**
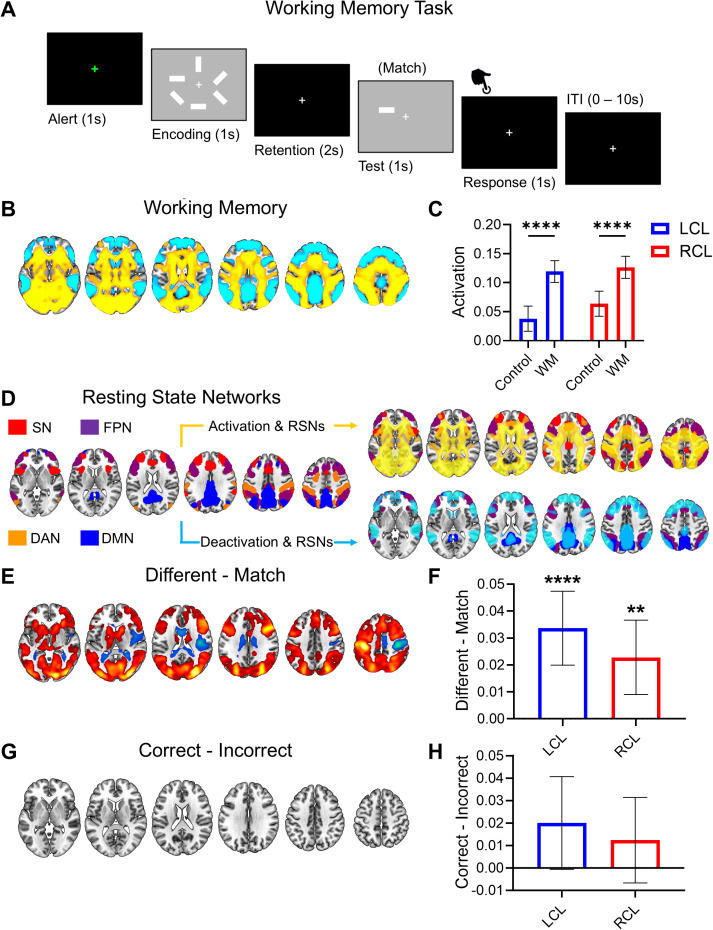
Claustrum co-activates with externally driven working memory networks. **(A)** Working memory trials comprised alert, encoding, retention, test, and response phases. **(B)** BOLD signal increases (warm) and decreases (cool) during working memory (*n* = 420). **(C)** Average parameter estimation (regression slope) of condition-related BOLD signal change detected significantly increased bilateral claustrum activity in working memory compared to control trials (two-way analysis of variance [ANOVA] main effect of condition: *F* (1, 838) = 147.7, *p* < 0.0001; post hoc LCL working memory vs. control: *p* < 0.0001; post hoc RCL working memory vs. control: *p* < 0.0001). No main effect of hemisphere (*F* (1, 838) = 1.536, *p* = 0.2156) or condition x hemisphere interaction (*F* (1, 838) = 2.415, *p* = 0.1206) were detected. **(D)** Salience (red), fronto-parietal (purple), dorsal attention (orange), and default mode (blue) networks from resting state group-ICA with working memory BOLD results overlaid. **(E)** “Different - match” trials contrast with greater signal during “different” in red and during “match” in blue. **(F)** Significantly greater LCL (*t* = 4.828, *p* < 0.0001) and RCL (*t* = 3.249, *p* = 0.0013) activi*t*y in “different” vs. “ma*t*ch” trials. **(G)** No brain-wide differences were observed when contrasting correct and incorrect working memory trials (*n* = 416). **(H)** No effects were observed in LCL (Wilcoxon Signed Rank Test *W* = 8,928, *p* = 0.0689) or RCL (*W* = 6,236, *p* = 0.2039) in “correct - incorrect” contrasts. Whole-brain responses were displayed on axial slice montages (z = 0, 10, 20, 30, 40, 50). Results were voxel-wise thresholded at *p* < 0.001, followed by family-wise error (FWE) cluster correction. Bar graphs display means with 95% confidence intervals. * represents *p* < 0.05, ** < 0.01, *** < 0.001, **** < 0.0001. Panel 1A is adapted with permission from Snoek and colleagues (2021) [29]. The data underlying bar graphs can be found in S1 Data.

We first sought to test if the claustrum activates with networks required for an externally driven task—working memory (Fig 1A)—in the Amsterdam Open MRI Collection (AOMIC) [29]. Two publicly available AOMIC datasets were analyzed (*n* = 198 and 222). When initial analyses in the first dataset were replicated in the second, the datasets were combined (S1–S5 Figs). Except where otherwise noted, all main text results were derived from the pooled sample (*n* = 420).

Compared to control trials with no working memory load, trials requiring working memory exhibited whole-brain maps typical for visual attention tasks (Fig 1B). For example, activity was observed in visual, insular, lateral frontal, and superior parietal cortices; while reduced activity was observed in ventromedial prefrontal and posterior cingulate cortices. Activity in left (LCL) and right (RCL) claustrum was also greater in working memory (WM) trials (Fig 1C). Brief, non-jittered intervals of task components necessitated analyzing entire trials (S1 Table), and claustrum blood oxygenation level dependent (BOLD) signal was not significantly correlated with motion (S2 Table).

Group Independent Component Analysis (ICA) of resting state scans from the same participants identified cognitive control-related networks: the salience network (SN) [30,31], fronto-parietal network (FPN) [32,33], dorsal attention network (DAN) [27,34], and default mode network (DMN) [35] (Fig 1D). The working memory task was associated with increased activity in salience (e.g., bilateral anterior insula, dorsal anterior cingulate cortex) and dorsal attention (e.g., posterior parietal cortices) network regions, and decreased activity in default mode regions (e.g., posterior cingulate cortex/precuneus) as expected.

During working memory trials, participants judged if a test stimulus was different from or matched the orientation of a rectangle in a previously presented array. Accuracy and reaction times did not differ between “different” and “match” trials (S3 and S4 Tables). However, the “different—match” contrast revealed differences in whole-brain responses. Correct responses to “different” trials were made with the right index finger and therefore activated left hemisphere motor cortex. Correct responses to “match” trials were made with the left index finger and so activated right hemisphere motor cortex. Although both trial types evoked bilateral claustrum and network responses (S3 Fig), “different” trials exhibited stronger intensity network signal compared to “match” trials (Fig 1E). Bilateral claustrum also exhibited significantly greater activity in “different” compared to “match” trials (Fig 1F), illustrating complementary increases in claustrum and task-dependent network BOLD signal.

When contrasting correct versus incorrect working memory trials (*n* = 416, 4 participants performed 0 incorrect trials; PIOP1 *n* = 195; PIOP2 *n* = 221), no differences were detected in whole-brain responses (Fig 1G) or claustrum activity (Fig 1H). Taking this together with the finding that stronger claustrum signal was paired with stronger intensity network signals (Fig 1E and 1F), increased task-associated network signal was always and only observed when claustrum signal increased.

### Claustrum co-activates with internally driven autobiographical memory networks

**Fig 2:**
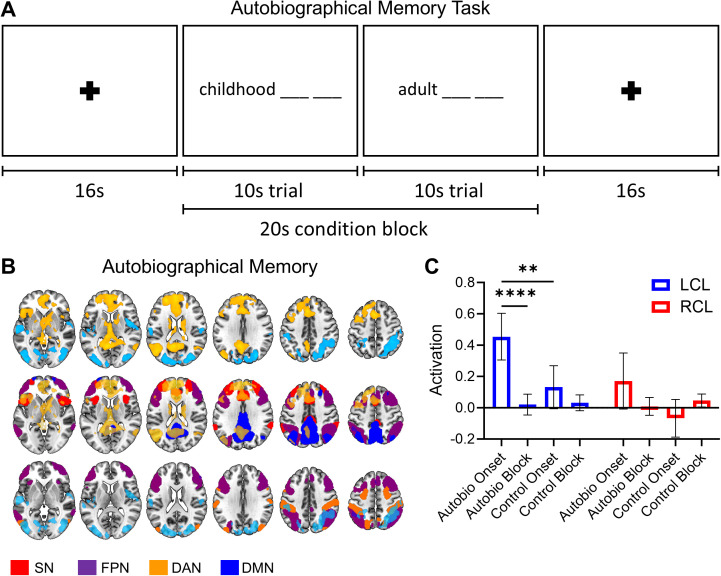
Claustrum co-activates with internally driven autobiographical memory networks. **(A)** Autobiographical memory scans consisted of 20 s blocks with 2 consecutive 10 s trials. Trials presented a time period label followed by a word pair either corresponding to a participant’s personal memory or not. **(B) Top:** BOLD signal increases (warm) and decreases (cool) during autobiographical memory (*n* = 35) **Middle:** BOLD signal increases from Top overlaid on salience, fronto-parietal, and default mode networks. Note task-related default mode network activity (e.g., PCC). **Bottom:** BOLD signal decreases from Top overlaid on fronto-parietal and dorsal attention networks. Displayed networks derived from AOMIC data due to absence of resting-state scans in the autobiographical memory dataset. **(C)** LCL, but not RCL, displayed significant activation at autobiographical memory (Autobio) trial onset (two-way ANOVA main effect of condition: *F* (2.129, 144.8) = 13.60, *p* < 0.0001; main effect of hemisphere: *F* (1, 68) = 9.227, *p* = 0.0034; condition x hemisphere interaction: *F* (3, 204) = 3.502, *p* = 0.0164; post hoc LCL Autobio onset vs. Autobio block: *p* < 0.0001; post hoc LCL Autobio onset vs. control onset: *p* = 0.0068). Bar graph displays means with 95% confidence intervals. The data underlying this panel can be found in S1 Data.

We next tested claustrum involvement in another task recruiting a different network phenotype: autobiographical memory. Although the default mode network is typically associated with task-induced deactivations, tasks with internally-directed demands have demonstrated increased default mode network activity [28,36–38] alongside other cognitive control networks. Analysis of a publicly available dataset (*n* = 35) [38] with fMRI scans from an autobiographical memory task (Fig 2A) confirmed the recruitment of a different network state than working memory, with activations in default mode (e.g., posterior cingulate cortex/precuneus) and salience (e.g., anterior insula, dorsal anterior cingulate cortex) network regions, and deactivations in dorsal attention (e.g., posterior parietal cortex, anterior supramarginal gyrus) network regions (Fig 2B).

A significant unilateral activation at the onset of autobiographical memory trials was observed in left claustrum. Analysis of task-onset responses was permitted by task timings (S5 Table), and left claustrum activity was not significantly correlated with subject motion (S2 Table). Greater left claustrum activity at autobiographical memory trial “onsets” relative to control trial onsets and to subsequent autobiographical memory trial “blocks” was consistent with cognitive-load dependent claustrum activity coinciding with the onset of task-associated network activity (Fig 2C).

Subsequent analyses focused on the left hemisphere, where claustrum activation was observed during working memory and autobiographical memory. The anterior insula and pulvinar, regions implicated in cognitive control network dynamics [39–41], also activated during both memory tasks (S6 and S7 Figs). Left anterior insula (LaINS) activity was less specific to the onset of autobiographical memory conditions than left claustrum, as it exhibited significant activity during autobiographical memory and control trials (S8 Fig). Left pulvinar (LPulv) activity during autobiographical memory was significantly correlated with subject motion (S6 Table).

### Claustrum is anatomically connected to cognitive task-associated network nodes

**Fig 3:**
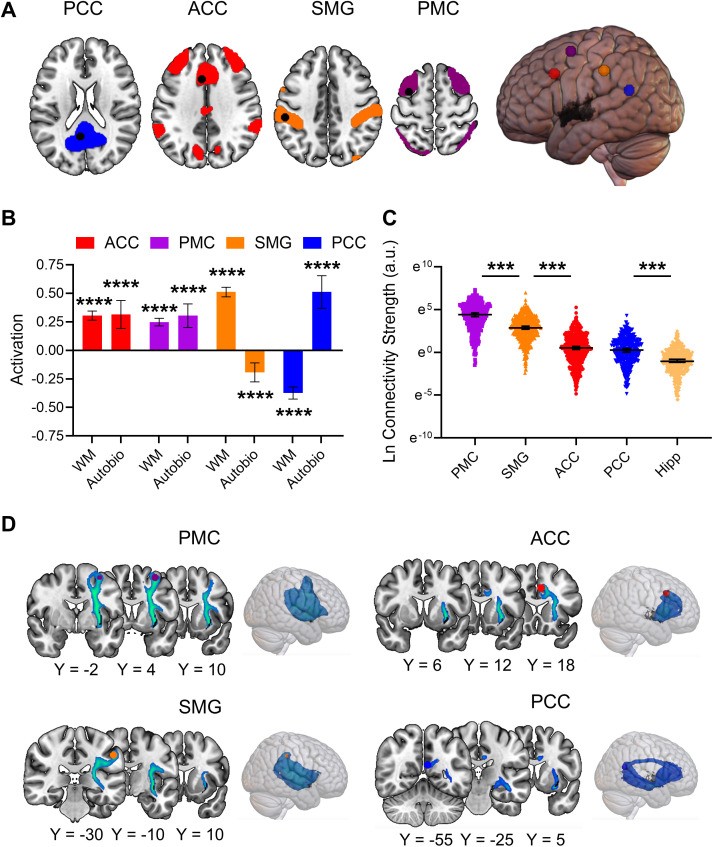
Claustrum is anatomically connected to cognitive task-associated network nodes. **(A)** Axial slices (*z* = 20, 35, 40, 60) individually displaying PCC, ACC, SMG, and PMC ROIs (black circles) on their respective networks, and rendered brain with LCL (black). **(B)** Activation parameters in “experimental - control” contrasts in working memory) and Autobio. All regions exhibited contrast values significantly different than zero (ACC WM: *t* = 15.15, false discovery rate (FDR) corrected *p* (*p*-FDR) < 0.0001; ACC Autobio: *t* = 5.21, *p*-FDR < 0.0001; PMC WM: *t* = 14.81, *p*-FDR < 0.0001; PMC Autobio: *t* = 5.98, *p*-FDR < 0.0001; SMG WM: *t* = 24.17, *p*-FDR < 0.0001; SMG Autobio: *t* = 4.71, *p*-FDR < 0.0001; PCC WM: *t* = 13.83; *p*-FDR < 0.0001; PCC Au*t*obio: *t* = 7.26, *p*-FDR < 0.0001). Bar graph displays means with 95% confidence in*t*ervals **(C)** LCL exhibited preferen*t*ial structural connec*t*ivity with PMC and SMG over ACC and PCC. Select comparisons shown for clarity. Plot displays medians with 95% confidence intervals. **(D)** Group tractograms for LCL connections with network representative nodes thresholded at 50%, meaning fibers detected in at least 50% of participants are shown. The data underlying panels (B) and (C) can be found in S1 Data.

Having observed increased claustrum activity during multiple task-dependent network states, we sought to characterize claustrum anatomical connectivity with task-associated network regions. We predicted left claustrum would possess structural connectivity with representatives of task-related cognitive control networks. The anterior cingulate cortex (ACC), premotor cortex (PMC), anterior supramarginal gyrus (SMG), and posterior cingulate cortex/precuneus (PCC) were chosen as representative regions of the salience, fronto-parietal, dorsal attention, and default mode networks, respectively (Figs 3A, 3B, and S9). See Materials and methods for region choice rationale. The hippocampus (Hipp) was used as a negative control, given evidence for sparse connectivity between the claustrum and hippocampus in humans and macaques [16,42]. All regions of interest (ROIs) were in the left hemisphere due to the ipsilateral bias for claustrum afferents and efferents in macaques [42] and humans [43].

Probabilistic tractography in AOMIC dMRI scans (*n* = 420) revealed significant differences in left claustrum structural connectivity with network representative regions (Fig 3C, *χ*^2^(4) = 1068.958, *p* < 0.001). Post hoc analyses showed that all claustrum connections with network representative regions were significantly stronger than the claustrum-hippocampus circuit (Fig 3C and 3D; *p*-FWE < 0.001). All structural connectivity *p*-values are shown in S7 and S8 Tables. Left claustrum also exhibited significant resting state functional connectivity with all identified cognitive control networks and all network representative regions (S9 Table).

Comparing median structural connectivity strengths (a.u.) of tractograms between claustrum and network representative regions revealed preferential claustrum structural connectivity with fronto-parietal and dorsal attention network representatives. The strongest claustrum connectivity was with the PMC (PMC > SMG: *z* = 6.568, *p*-FWE < 0.001), followed by SMG (SMG > ACC: *z* = 12.962, *p*-FWE < 0.001; SMG > PCC: *z* = 14.882, *p*-FWE < 0.001), and finally ACC and PCC, which did not exhibit significantly different claustrum connectivity (ACC > PCC: *z* = 1.92, *p*-FWE = 0.548). Left claustrum also exhibited significantly greater structural connectivity in this dataset than left anterior insula with all network representative regions, and greater structural connectivity than left pulvinar with PMC, SMG, and ACC (S8 Table). These findings are consistent with macaque tracer evidence of greater claustrum than anterior insula anatomical projection strength broadly across the cortex, including premotor, temporoparietal, and anterior cingulate regions [42].

### Effective connectivity suggests excitatory claustrum influence on multiple task-related network regions

Given structural data corroborating claustrum connectivity with cognitive control network regions, we devised a circuit model to investigate how claustrum activity influences, and is influenced by, network region activity. We tested if any representative network regions were task-dependent sources of left claustrum input or targets of left claustrum output. In mice, ACC input to the claustrum is necessary for optimal performance in a cognitively demanding task [21], and the claustrum robustly relays ACC input to frontal and posterior cortical regions [20]. Therefore, we predicted that ACC would supply driving input to the human claustrum, which would exert excitatory influence on task-related regions.

**Fig 4:**
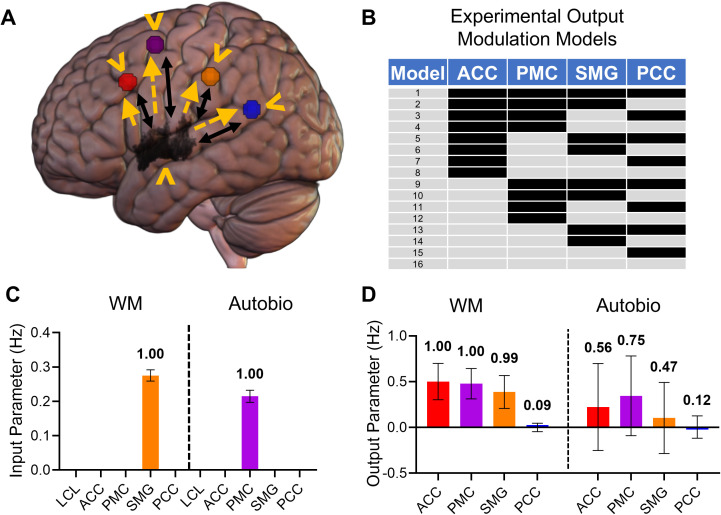
Effective connectivity suggests excitatory claustrum influence on multiple task-related network regions. **(A)** The “full” DCM contained all verified anatomical connections (black arrows), possible driving experimental input (orange ‘^’ symbols) from each node, and possible task-induced modulation of all LCL outputs (orange dashed arrows). **(B)** Tested models represented all 16 possible combinations of experimental claustrum output modulation from a full model (#1) to a null model (#16). **(C) Left:** Estimated DCM input parameters during WM and Autobio **(D)** Estimated DCM output parameters during WM and Autobio. 95% confidence intervals and posterior probabilities signifying evidence strength (>0.5 = “weak”, >0.75 = “positive”, >0.95 = “strong”, >0.99 = “very strong”) accompany bars. The data and code used to produce bar graphs can be found in https://osf.io/akps6.

Dynamic causal models (DCM) [44] were defined ranging from a full model in which memory tasks modulated all claustrum projections to a null model in which no claustrum projections were task-responsive (Fig 4A and 4B). This set of models was crossed with five possible sources of claustrum input, including the four representative regions of networks of interest: ACC (salience), PMC (fronto-parietal), SMG (dorsal attention), PCC (default mode); and the claustrum itself, which represented all other potential input sources. Left anterior insula and left pulvinar effective connectivity with network representative regions were also analyzed (S6 and S7 Figs).

Positive input parameters and high posterior probabilities indicated very strong evidence for SMG input to left claustrum during working memory and PMC input during autobiographical memory (Fig 4C). Comparisons between tasks revealed very strong evidence for greater SMG input to claustrum during working memory (S10 Fig). Leave-one-out cross-validation revealed that prediction of task state (working memory versus autobiographical memory) using claustrum input parameters was statistically significant (S10 Table). Left anterior insula and left pulvinar input parameter results were strikingly similar to left claustrum, raising the possibility that DCM input parameter estimates were driven primarily by activity in modeled sources of input to analyzed regions rather than activity in the regions themselves (S10 and S11 Tables)

Task-related output parameters reflected strong or very strong evidence for excitatory left claustrum effective connectivity to ACC, PMC, and SMG, but not PCC during working memory, as well as weak evidence for excitatory left claustrum influence on ACC and PMC during autobiographical memory (Fig 4D). Comparisons between tasks revealed weak evidence for greater left claustrum excitatory influence on ACC and SMG in working memory (S9 Fig), and output parameters were unable to predict task state. Leave-one- out cross validation was similarly unable to predict task state from left anterior insula and left pulvinar output parameters. Unlike input parameters, output parameters exhibited qualitative differences between left claustrum, anterior insula, and pulvinar. Leave-one-out cross-validation was performed to see if output parameters were significantly different between these seed regions. When comparing left claustrum and left anterior insula, working memory output parameters significantly distinguished the regions, and autobiographical memory output parameters exhibited a trend toward significance after correcting for multiple comparisons despite the substantially smaller sample size in that task. Output parameters did not reliably distinguish between left claustrum and left pulvinar. All output parameter cross-validation results and Bayesian output model comparison probabilities are shown in S10 and S12 Tables, respectively.

Model parameters suggested left claustrum exerted excitatory influence on SMG, a dorsal attention network region, during externally driven working memory but not autobiographical memory. PMC, a fronto-parietal network region, was identified as a possible recipient of claustrum output in both tasks, consistent with a fronto-parietal network role in integrating task-dependent network components [36,45]. The ACC, a salience network region, was also estimated to receive excitatory influence from left claustrum during both tasks, with stronger evidence of this during working memory. No analyses supported left claustrum effective connectivity with PCC, a default mode network region.

## Discussion

Our results implicate the claustrum in multiple network states associated with diverse cognitive demands. Analyses are consistent with the claustrum exerting task-responsive excitatory influence on regions representing multiple cognitive control networks during externally and internally driven tasks. Findings also indicate preferential claustrum anatomical connectivity with fronto-parietal and dorsal attention over salience and default mode network regions. Comparisons with the anterior insula and pulvinar support the specificity of these findings to the claustrum and establish this long-understudied structure as a key player in task-dependent network dynamics underlying cognitive control.

### Claustrum effects coincide with cognitive control network changes

Findings from both memory tasks, which evoked starkly different network motifs, implicate the claustrum in a wide range of cognitive control network states. Notably, increases in claustrum activity aligned with increases in task-dependent network activity. The working memory task included conditions when the test stimulus was different from or matched an encoded stimulus. “Different” and “match” trials evoked similar whole-brain activity maps, but overall BOLD signal intensity was greater during “different” trials. Analogously, claustrum activity was observed in both conditions, and claustrum activity was significantly greater in “different” than “match” trials. Conversely, no differences were detected in brain-wide or claustrum signal between correct and incorrect trials. Therefore, whole-brain network effects always and only appeared alongside claustrum effects.

Previous work implicates the claustrum in motor planning [46]. The evidence from this study, while in no way negating those findings, links claustrum activity to non-motor cognitive processes. The working memory “different” versus “match” condition effects mentioned above must have arisen after the test stimulus appeared: while processing the test stimulus, executing the motor response, or both. “Different” and “match” trials required button presses from opposite hands, which was evident in opposing unilateral motor cortex responses, but increased claustrum activity was bilateral in both conditions. Moreover, bilateral claustrum signal was greater in “different” than “match” trials despite comparable motor demands. Cognitive control network and claustrum BOLD activity likely also occurred in earlier task phases, such as stimulus encoding and retention, a claim supported by similar patterns of network activity and increased bilateral claustrum signal when working memory trials were compared to control trials, which lacked working memory demands yet included motor responses. Left claustrum signal also increased at the onset of autobiographical memory trials, during a task with no motor response. These results support claustrum participation in task-dependent networks states subserving cognitive control.

### Structural and functional claustrum-network relationships

dMRI analyses revealed tiers of claustrum structural connectivity. Claustrum-hippocampus connectivity was the weakest analyzed, consistent with findings in macaques and humans motivating its use as a negative control [16,42]. Claustrum connections with ACC and PCC, representative nodes of salience and default mode networks, were significantly stronger than claustrum-hippocampus, and claustrum connections with SMG and PMC, representatives of dorsal attention and fronto-parietal networks, were the strongest observed. This did not match our expectation that ACC would possess the strongest claustrum connectivity given the strength and functional importance of claustrum-ACC connections in rodents [19,21–23,47–50]. It is possible this resulted from measurement error. dMRI tractography connection weights correlate with those derived from tracer injections, but the method is not perfect [51]. However, these findings may also reflect species differences due to significant human cortical expansion [52]. Indeed, recent macaque tracing evidence indicates that claustrum-anterior cingulate connectivity is strong but not dominant across the cortex in terms of claustrum input or output [42].

Preferential claustrum connectivity with dorsal attention and fronto-parietal networks is supported by a previous claustrum dMRI analysis [53] and by this study’s DCM, which found evidence for excitatory claustrum influence on PMC in the fronto-parietal network during both tasks and on SMG in the dorsal attention network during working memory. DCM supported excitatory claustrum influence on ACC in the salience network in both tasks as well, but the absence of claustrum-PCC effective connectivity in the default mode network is conspicuous and reinforces the general agreement between DCM and dMRI, which found relatively weak claustrum-PCC structural connectivity.

The effect of claustrum projections on their targets vary based on several factors [54], and we have speculated previously on the possibility of claustrum interactions with the default mode network [26]. While we predicted claustrum supported task-dependent network activity, we were uncertain if that entailed excitatory influence on task-related regions, inhibitory influence on deactivated regions, or both. DCM suggested that the claustrum exerts task-dependent excitatory influence on ACC, PMC, and SMG, representatives of multiple cognitive control networks. No evidence of claustrum influence on PCC was found, so the functional relevance of the observed claustrum-PCC structural connection may apply in other brain states, such as rest or sleep [55]. Importantly, although we did not observe evidence for inhibitory claustrum influence on network regions at rest, the claustrum excitatory influence on network regions is consistent with task-dependent increased excitation or reduced inhibition.

### A distinct claustrum role in task-dependent network states

Control analyses of the pulvinar and anterior insula revealed different results than the claustrum. The pulvinar nuclei connect with prefrontal and posterior parietal cortices associated with cognitive control networks [56–58], and task-based fMRI has implicated the region in working memory [59]. Consistent with this literature, we observed increased medial pulvinar BOLD signal during working memory, but evidence for pulvinar activity during autobiographical memory was less robust. A significant BOLD signal increase at autobiographical memory trial onset may have been artifactual as it shared a significant positive correlation with subject movement. Moreover, DCM evidence for excitatory pulvinar output during autobiographical memory was comparable to that of the null model, which contained no task-induced modulation of pulvinar projections. Lastly, pulvinar exhibited preferential structural connectivity with the hippocampus, the negative control region for claustrum structural connectivity analyses. These findings are consistent with the pulvinar performing a different role than claustrum in cognitive control network coordination, and with that role being more relevant in the externally driven working memory task.

The anterior insula shares connections with cognitive control-associated cortical regions [60,61] and responds to a wide variety of stimuli prior to other brain regions [62]. Experimental support has even been found for a causal influence of anterior insula activity on cognitive control network regions; however, this evidence has been shown in relatively simple tasks [62]. Analyses of anterior insula effective connectivity specific to conditions of increased cognitive demand find causal influence restricted to the dorsal anterior cingulate, the anterior insula’s salience network counterpart [63]. This literature is consistent with anterior insula recognizing cognitive control needs and downstream mechanisms contributing to demand-dependent network activation. We observed significant anterior insula BOLD increases during both memory tasks. Notably, anterior insula responses during autobiographical memory scans extended into trial “blocks” and were also detected in response to control stimuli, whereas claustrum effects were observed specifically at the onset of autobiographical memory trials. Surprisingly, DCM found strong evidence for inhibitory influence of anterior insula outputs on network representative regions. Given contrary findings in prior studies using different effective connectivity techniques, the limitations of DCM prevent us from concluding anterior insula projections are inhibitory. Importantly, effective connectivity models were statistically significant in their ability to predict if task-dependent output parameters were associated with anterior insula or claustrum. Distinct task response profiles, as well as significantly different structural and effective connectivity with network representative regions in the analyzed datasets, are consistent with the anterior insula and claustrum performing different, likely complementary, roles in network mechanisms of cognitive control.

### Limitations

This study’s findings are subject to important limitations. Bilateral claustrum responses were observed during working memory, but only left claustrum responses were significant during autobiographical memory. This may be due to differences in sample size and handedness between the datasets, or due to differences in task modeling. However, task differences in claustrum effects could have arisen from laterality in claustrum function, a possibility increasingly evinced in other human claustrum studies [18,53,64]. Our focus on task differences restricted circuit analyses to the left hemisphere, where claustrum effects were observed in both tasks, leaving laterality in structural connectivity and effective connectivity unexplored.

Structural connectivity measures are data-derived inferences of axonal pathways, not a literal count of white matter tracts. Consequently, measures are impacted by such factors as connection distance and the presence of differentially oriented fibers within voxels [51,65]. Also, tractography analyses could not reveal the relative contributions of afferent and efferent projections to claustrum structural connectivity because dMRI is agnostic to the direction of fibers.

Our effective connectivity analyses pertained to region-level effects among key representatives of large-scale brain networks, not network-level effects. Therefore, network-level interpretations of effective connectivity can only be inferential. Additionally, although other connections and regions are undoubtedly involved in mechanisms of cognitive control network activity, we opted to focus effective connectivity model complexity on circuit aspects relevant to this study’s research questions: inputs to, and outputs from, the claustrum.

Lastly, imaging the human claustrum at 3T entails limits in spatial specificity that impact all analyses. Signal regression from neighboring regions and comparisons with functionally related regions are consistent with claustrum specificity in this study, but the analyses are still vulnerable to partial volume effects. Replication of these and other human claustrum neuroimaging findings at 7T would be valuable.

### Conclusions

Significant claustrum responses were observed to externally and internally driven cognitive control demands, which evoked markedly different network states. The claustrum also exhibited significant structural connectivity with cognitive control network regions and displayed excitatory effective connectivity with regions representing multiple active networks across tasks. Control analyses revealed distinct task response, anatomical connectivity, and effective connectivity profiles for the claustrum, anterior insula, and pulvinar, suggesting distinct roles for these regions in modulating task-dependent network dynamics. These findings converge to implicate the claustrum in supporting task-dependent activity in multiple, large-scale cognitive control networks.

## Materials and methods

### Participants

Analyses were performed in three datasets. The first two datasets are components of the publicly available, multimodal Amsterdam Open MRI Collection (AOMIC; https://nilab-uva.github.io/AOMIC.github.io/). Full details on participant inclusion and demographic variables can be found in Snoek and colleagues (2021) [29]. Briefly, datasets labeled PIOP1 and PIOP2 consisted of university students at the Amsterdam University of Applied Sciences and the University of Amsterdam. Of the manifold AOMIC scans available, this study analyzed working memory task, resting-state, and dMRI scans. Because structural and effective connectivity analysis ROIs were selected based on task responses and resting state network membership, all analyses were performed on the subset of participants who possessed all three scan types (PIOP1: *n* = 198, 108 female, 83 male, 7 unspecified, mean ± sd. age = 22.20 ± 1.81 years; PIOP2: *n* = 222, 126 female, 95 male, 1 unspecified, mean ± sd. age = 21.95 ± 1.79 years). Of the 216 PIOP1 participants, 18 were excluded due to the absence of a desired scan. Of the 226 PIOP2 participants, 4 were excluded due to the absence of a desired scan. When initial analyses of PIOP1 were reproduced in PIOP2 (S1–S5 Figs), the datasets were combined. Except where otherwise noted, all main text results were derived from the pooled sample (*n* = 420).

The third dataset was obtained by Fuentes-Claramonte and colleagues (2019) [38] and was selected for this study for analysis of an autobiographical memory task (https://openneuro.org/datasets/ds001618/versions/1.0.1). After the exclusion of 1 participant due to excessive motion, this dataset yielded *n* = 35 (mean ± sd. age = 40.89 ± 12.02 years, all right-handed).

The authors of both studies obtained approval from their overseeing ethics committees prior to data acquisition, and all participants provided written informed consent. Snoek and colleagues (2021) [29] received approval from the University of Amsterdam’s ethical committee, and Fuentes-Claramonte and colleagues (2019) [38] received approval from the Clinical Research Ethics Committee of the Sisters Hospitallers (Comité de Ética de Investigación Clínica de las Hermanas Hospitalarias).

### Imaging acquisition and preprocessing

#### AOMIC.

Full details of scan acquisition can be found in Snoek and colleagues (2021) [29]. Both PIOP1 and PIOP2 datasets were acquired on the same Phillips 3T scanner (Philips, Best, the Netherlands) with a 32-channel head coil. PIOP1 was acquired on the “Achieva” version, and PIOP2 was acquired later, after the scanner was upgraded to the “Achieva dStream” version. Both datasets acquired high-resolution 3D MPRAGE anatomical scans (repetition time/echo time [TR/TE] 8.5/3.9ms, slice thickness 1 mm, field of view [FOV] 188 × 240 × 220 mm, flip angle 8°, voxel size 1 × 1 × 1 mm).

All AOMIC data was standardized in Brain Imaging Data Structure format and preprocessed with Fmriprep version 1.4.1 (RRID:SCR_016216) [66,67], a Nipype based tool (RRID:SCR_002502) [68,69].

#### *Working memory*.

PIOP1 and PIOP2 working memory functional data were acquired with a gradient echo echo-planar imaging (EPI) sequence (TR = 2,000 ms, TE = 28 ms, 37 slices, FOV = 240 × 240 × 122 mm, flip angle = 76.1°, voxel size 3 × 3 × 3 mm, slice gap = 0.3 mm).

Publicly available preprocessed functional images were unsmoothed. Additional preprocessing was performed in SPM12 (https://www.fil.ion.ucl.ac.uk/spm/software/spm12/). Images were resampled to 2 × 2 × 2 mm voxels with a 4th degree B-Spline interpolation. Claustrum analyses used unsmoothed images to prevent inclusion of extra-claustral signal in claustrum ROI voxels. All other analyses used images smoothed with a 6 mm full-width at half maximum (FWHM) Gaussian kernel.

#### *Resting-state*.

PIOP1 resting state data were acquired with a gradient echo EPI sequence (TR = 750 ms, TE = 28 ms, 36 slices, FOV = 240 × 240 × 118 mm, flip angle = 60°, voxel size 3 × 3 × 3 mm, slice gap = 0.3 mm). PIOP2 resting state data were acquired with a gradient echo EPI sequence (TR = 2,000 ms, TE = 28 ms, 37 slices, FOV = 240 × 240 × 122 mm, flip angle = 76.1°, voxel size 3 × 3 × 3 mm, slice gap = 0.3 mm). PIOP1 resting state scans lasted 6 min, and PIOP2 resting state scans lasted 8 min. PIOP1 resting state scans were acquired with multi-slice acceleration (factor 3), while PIOP2 scans were not, resulting in different TRs.

Publicly available preprocessed functional images were unsmoothed. Additional preprocessing was performed in in SPM12 (https://www.fil.ion.ucl.ac.uk/spm/software/spm12/). Images were resampled to 2 × 2 × 2 mm voxels with a 4th degree B-Spline interpolation and smoothed with a 6 mm FWHM Gaussian kernel.

Participants were directed to maintain their gaze on a fixation plus sign in the middle of a screen and to let their thoughts run freely.

#### *Diffusion weighted imaging*.

PIOP1 and PIOP2 used spin-echo diffusion imaging over a single run (TR = 7,387 ms, TE = 86 ms, slice thickness = 2 mm, FOV = 224 × 224 × 120 mm). Diffusion weighting consisted of a single shell (*b* = 1,000 s/mm^2^). Each scan contained 32 diffusion-weighted directions, and a single non-diffusion-weighting image (b0).

Preprocessed dMRI scans were downloaded from the AOMIC database, which underwent a custom preprocessing pipeline utilizing tools from both FSL (the FMRIB Software Library) [70] and MRtrix3 [71]. MRtrix3 was used for denoising (dwidenoise) [72,73], Gibbs artifact removal (mrdegibbs) [74], eddy current and motion correction (dwipreproc), which utilizes FSL’s eddy [75], bias correction (dwibiascorrect) [71], brain mask extraction (dwi2mask) [76], and gradient diffusion correction (dwigradcheck) [77]. After preprocessing, a diffusion tensor model was fit to the data (dwi2tensor) [78], a fractional anisotropy image was extracted (tensor2metric) [79], and finally, a population fractional anisotropy template was created (population_template).

#### *Autobiographical memory*.

Full details of scan acquisition can be found in Fuentes-Claramonte and colleagues (2019) [38]. Autobiographical memory scans were acquired with a 3T Philips Achieva scanner (Philips Medical Systems, Best, the Netherlands). Functional data were acquired with a T2*-weighted EPI sequence (TR = 2,000 ms, TE = 30 ms, slice thickness = 3 mm, FOV = 240 mm, flip angle = 78°, in-plane resolution = 3 × 3 mm, inter-slice gap = 1 mm). Thirty-two slices were acquired per volume with an ascending order parallel to the AC–PC plane. Fuentes-Claramonte and colleagues (2019) [38] discarded the first 10 volumes to avoid T1 saturation effects. In this study, all volumes were included because no differences were noted in claustrum signal when discarding or including initial volumes. If anything, claustrum response values marginally increased when discarding initial volumes, potentially biasing results toward significant effects (S11 Fig).

All autobiographical memory scans were preprocessed in SPM12 (https://www.fil.ion.ucl.ac.uk/spm/software/spm12/). Preprocessing included realignment (motion correction) and coregistration and normalization of realigned functional images to SPM’s template mean EPI image file [80] in Montreal Neurological Institute (MNI) space with interpolation to 2 × 2 × 2 mm voxels using a 2nd degree B-Spline. Claustrum analyses used unsmoothed images to prevent inclusion of extra-claustral signal in claustrum ROI voxels. Whole-brain task response analyses used images smoothed with a 6 mm FWHM Gaussian kernel.

### Tasks

#### Working memory.

The working memory task (Fig 1A) was designed to measure brain processes related to visual working memory [29] using a protocol inspired by Pessoa and colleagues (2002) [81]. Each run consisted of 40 trials: 32 trials which possessed a working memory load, and 8 trials which did not, but were designed to control for visual stimulation and motor responses.

During the inter-trial interval (ITI), participants attended to a white fixation plus sign on a black background. Trials began with a change of the plus sign’s color from white to green, which lasted for 1 s, alerting the participant to trial onset (“alert” phase). In working memory trials, this was followed by a change in background color from black to gray and the presentation of an array of 6 white bars arranged in a circle with random orientations, which appeared for 1 s (“encoding” phase). Control trials only included the change in background color. After this, the white fixation plus sign was displayed on the original black background for 2 s (“retention” phase). Subsequently, working memory trials displayed a gray background with a test stimulus of one bar (“test” phase). After 1 s of test stimulus presentation, the background returned to black, and participants had 1 s to respond if the test stimulus matched (16 trials, left index finger press) or differed from (16 trials, right index finger press) a bar in the trial’s previously displayed array (“response” phase). Instead of displaying a test stimulus, control trials similarly changed background from black to gray, but displayed text directing the participant to “respond left” or “respond right”. After 1 s, these visual instructions disappeared, the background returned to black, and participants had 1 s to button-press with the directed finger.

#### *Autobiographical memory*.

Immediately prior to the fMRI session, a member of the data-acquiring research team [38] interviewed each participant for approximately 1 hour. The interview consisted of prompts from the Autobiographical Memory Interview (AMI) [82] and the Crovitz test [83] to identify four to six memories from each of four time periods: “childhood” (before age 12), “adolescence” (ages 12–18), “adulthood” (after age 18), and “recently” (within the last year). Autobiographical memory stimuli were three-word prompts, comprising a time period label and a word pair signifying a previously identified memory. Word pairs were decided collaboratively during the interview by the researcher and participant, and word pairs were only generated for memories receiving a maximum AMI score of 3 for the memory’s level of detail and specificity in time and place. Control stimuli, designed to align with autobiographical memory stimuli in terms of perceptual and linguistic processing [38], were also three-word prompts, but comprising a time period label followed by a word pair with no reported memory association. Scans consisted of 20 stimulation blocks separated by 16 s long ITIs where a fixation plus sign was presented (Fig 2A). Blocks alternated between control stimuli and autobiographical stimuli, and each 20 s block consisted of 2 trials, each lasting 10 s. Participants were directed to read the words presented at each trial silently and recall any associated memory.

### Task modeling

Previous analyses of task-induced claustrum BOLD signal change have used hybrid event-block designs [17,53] where single condition blocks were modeled with two regressors, one representing a brief condition “onset” period and another representing the remainder of the condition “block”. This modeling format has tested the prediction that transient claustrum activation occurs at task onset to support task-associated network initiation, and claustrum BOLD signal increases have been observed specifically at the onset of a difficult cognitive task condition [17] and the onset of acute pain [53].

#### Working memory.

Although models representing each phase of the working memory task (i.e., alert, encoding, retention, test, response) were desired, the brief duration (1–2 s) and lack of jitter of the trial components resulted in highly correlated regressors and a high likelihood of effect misattribution (S1 Table). Therefore, working memory scan general linear models (GLMs) contained three regressors, representing the entire duration of each trial type (“working memory different,” “working memory match,” “control”), capturing ITI in the implicit baseline. For DCM analyses (see below), a second model with two regressors (“experimental,” “control”) was used where “working memory different” and “working memory match” were pooled into one “experimental” condition. Results from this model are presented in Fig 1B.

#### *Autobiographical memory*.

Low Variance Inflation Factors confirmed hybrid event-block GLMs were appropriate for the autobiographical memory task [84] (S5 Table). Therefore, each 10 s trial (2 per block) was modeled separately as a 2 s “onset” condition beginning at the trial onset followed by an 8 s “block” condition lasting the remainder of the trial. Separate “onset” and “block” regressors were used for autobiographical memory and control trials. As in the working memory task, the ITI was captured in the implicit baseline. For DCM analyses (see below), a second model with two regressors (“experimental,” “control”) was used where “onset” and “block” were combined yielding 10 s regressors lasting the duration of each trial.

All task fMRI analyses were performed in SPM12. For analyses of ROIs other than the claustrum, GLMs included all task conditions and six motion parameters. Second-level results were separately masked with each ROI and activation values were averaged across the ROI within each subject, and then across subjects. Separate GLMs were used to analyze each hemisphere’s claustrum ROI, which included all task conditions, six motion parameters, and the two flanking region-condition interaction timeseries generated via the task-adapted SRCC process described below.

### Small region confound correction

As described previously [17], the effects of neighboring insular cortex and putamen on claustrum signal were controlled via Small Region Confound Correction (SRCC). Insular cortex and putamen “flanking” ROIs were defined by dilating each hemisphere’s claustrum ROI four functional voxels and identifying the overlap between the dilated claustrum and the neighboring insula and putamen at least two functional voxels separated from the original claustrum. This generated “flanking” ROIs within the insular cortex and putamen, similar to the claustrum’s shape, yet apart from the claustrum to avoid including claustrum signal.

In previous claustrum resting state connectivity analyses [17], the effects of flanking regions were controlled by including their time series as additional regressors. However, if flanking ROI signals are influenced by task conditions, using physiological flanking ROI time series as additional regressors in task-based claustrum analyses risks removing condition-induced variation from the claustrum signal [53]. Therefore, when analyzing claustrum task responses, the CONN Toolbox (RRID:SCR_009550) [85] was used to generate canonical hemodynamic response function (HRF) convolved condition time series, and a regressor was generated for each flanking ROI by obtaining the de-meaned element-wise product of the ROI’s de-meaned physiological time series and the de-meaned, summed HRF-convolved time series of all modeled conditions. This yielded a time series for each flanking ROI that covaried with the ROI physiological time series during task conditions but lacked variation potentially induced by the conditions, allowing control of the influence of neighboring regions on claustrum signal without indirectly removing task effects.

### Resting state functional connectivity

Cognitive control networks were derived from PIOP1 and PIOP2 preprocessed, smoothed resting state scans in the CONN Toolbox (RRID:SCR_009550) [85]. Nuisance regressors included motion parameters and their first order derivatives, a scrubbing vector generated by ART-toolbox identification of outlier scans (global-signal z-value threshold = 5; subject-motion mm threshold = 0.9), the first five principal components of white matter and cerebrospinal fluid (CSF) masks (aCompCor) [86,87], and a default regressor in the CONN Toolbox to control for potential ramping effects at scan onset. Global signal regression was not performed. Consequently, a 2× eroded CSF mask and a 4× eroded white matter mask were used because such masks no longer contain global signal [88]. Regression was performed simultaneously with band-pass filtering [89] between 0.008 Hz and 0.09 Hz, and linear detrending was also performed.

Group-ICA was performed in the CONN Toolbox following methods described in Calhoun and colleagues (2001) [90] using individual subject dimensionality reduction to 64 dimensions prior to G1 FastICA and GICA3 Back-projection. Networks were selected from resulting spatial components based on comparisons with relevant literature [27,30–35,91] and the CONN Toolbox’s “spatial match to template” tool, which calculates component correlations with CONN’s default network atlas, derived from a group-ICA of Human Connectome Project data (*n* = 497). PIOP1 was subjected to a 10 component parcellation, which yielded all desired networks (salience, fronto-parietal, dorsal attention, default mode). However, a 10-component ICA did not robustly capture all desired networks in PIOP2 or when combining the datasets. Therefore, a 15-component ICA was used to define networks in PIOP2 specifically and when combining the datasets.

ROI-ROI resting state functional connectivity was also calculated in the combined AOMIC PIOP1&2 dataset using the CONN Toolbox. ROIs included LCL, the group-ICA derived networks, and the network representative regions described in “Regions of Interest” below. A ROI–ROI connectivity matrix was calculated with Fisher-transformed bivariate correlations between each pair of ROI BOLD time series. Participant-level Fisher-transformed *z* values were compared to 0 with *t*-tests to assess statistical significance.

### Regions of interest

All ROIs are publicly available on NeuroVault (https://neurovault.org/collections/16667/).

LCL, RCL, insular cortex, and putamen ROIs were drawn by hand on the normalized anatomical images of 20 subjects in a publicly available dataset (*n* = 22) acquired on a 7T MR Scanner (MAGNETOM 7T, Siemens Healthcare, Erlangen, Germany), and a group average ROI file was obtained for each region. Further details of the scans can be found in Gorgolewski and colleagues (2015) [92]. Two subjects were omitted from ROI generation, one due to preprocessing errors preventing normalization of sufficient quality and one due to acquisition via different scanning parameters. The relevance of insular cortex and putamen ROIs, as well as a description of “flanking” ROIs can be found in the “Small Region Confound Correction” Methods above. All SRCC-related ROIs are publicly available.

Circuit analysis ROIs were selected based on the overlap of “experimental - control” trial contrast maps (working memory: = experimental, control = control; autobiographical memory: autobiographical = experimental, control = control) and group-ICA resting state network maps. All ROIs were in the left hemisphere because only LCL exhibited significant responses to both memory tasks, and because recent tracing experiments in macaques observed a strong ipsilateral bias for both claustrum afferents and efferents [42], a finding supported by recent tractography analyses in humans [43].

Salience network activity appears in response to a wide variety of tasks and has been observed to temporally precede activity in other network regions [62]. It has therefore been proposed to “gate” the switching between default mode and task-positive network states [39,40]. ACC is a primary salience network node, and increasing evidence attests to the functional importance of bidirectional ACC-claustrum projections [21–23]. We therefore selected a salience network ROI in ACC (center: −6, 18, 36) that was active in both memory tasks.

A network of brain regions consistently active across a wide variety of cognitive control tasks has been referred to as the multiple demand network [93,94], and this network has been shown to consist primarily of fronto-parietal network regions [45]. We therefore selected an ROI active in both tasks that was centered within the data-derived fronto-parietal network and a publicly available multiple demand network map (https://imaging.mrc-cbu.cam.ac.uk/imaging/MDsystem). This ROI resided in PMC (center: −32, 2, 58).

The posterior cingulate cortex/precuneus (PCC) is a hallmark default mode network node [35], and the memory tasks were selected due to their predicted opposing effects on DMN activity. Therefore, a PCC ROI (center: −6, −54, 20) within the data-derived default mode network that exhibited differential activation between memory tasks was selected. Conversely, the dorsal attention network is characterized by its anticorrelation with the default mode network [34,95]. Therefore, an ROI from the AOMIC-derived dorsal attention network exhibiting a task response profile opposing that of PCC was selected. This ROI resided in SMG (center: −54, −30, 40).

Circuit analysis ROIs are shown overlaid on resting state networks in Fig 3A and in more detail in S9 Fig.

Left anterior insula location (center: −38, 18, −4) was selected in an area responsive to both memory tasks, similarly to ACC, as a salience network representative.

To define the left pulvinar ROI, a publicly available functional pulvinar parcellation [96] derived from a previous anatomical parcellation [97] was analyzed for memory task responses. The working memory task evoked relatively uniform responses across pulvinar parcels, whereas the autobiographical memory task evoked signal increases from ventromedial and dorsomedial parcels. These two parcels were combined and masked with SPM12’s cerebrospinal fluid tissue map due to the region’s proximity to ventricles. Human tractography confirms significant ipsilateral pulvinar structural connectivity [58].

For LCL structural connectivity analyses, a negative control region was selected in the left hippocampus (Hipp; center: −26, −20, −16) based on evidence of weak claustrum-hippocampus anatomical connectivity [16,42].

All circuit analysis ROIs except left pulvinar were generated in MarsBaR software [98] as 5 mm radius spheres.

### Structural connectivity

AOMIC dMRI scans underwent fiber tract estimation and orientation using FSL’s Bayesian Estimation of Diffusion Parameters Obtained using Sampling Techniques-Crossing Fibers (BEDPOSTx) [99,100].

Probabilistic tractography was run using FSL’s Probtrackx2 GPU version [99–101] to assess structural connectivity of LCL, LaINS, and LPulv with ACC, PMC, SMG, PCC, and Hipp. Each ROI was in MNI152 space and was non-linearly transformed to each individual’s diffusion space using FMRIB’s Non-linear Image Registration Tool (FNIRT) [70], during Probtrackx2 analysis. For each connection (e.g., LCL-ACC), two tractograms were computed, one for each direction, to control for directional biases in dMRI acquisition [102] and fiber fanning [103]. The modified Euler algorithm was used to generate the tracts with 10,000 streamlines per voxel. To exclude spurious connections, we included exclusion masks that guided tractography. All tractogram analyses used the following exclusion masks:

i. a combination of ACC, PMC, SMG, PCC, and Hipp masks depending on the analyzed target ROI (e.g., when assessing LCL-ACC connectivity, PMC, SMG, PCC, and Hipp exclusion masks were used),
ii. a combination of LCL, thalamus [104–107], and insular cortex [108] masks, depending on the analyzed seed ROI (i.e., LCL analyses used the insular cortex and thalamus masks, LaINS analyses used LCL and thalamus masks, and LPulv analyses used LCL and insular cortex masks),
iii. an amygdala mask [104–107],
iv. a mid-sagittal mask, to remove tracts crossing the midline, and
v. target-specific coronal and/or transverse plane masks to further constrain connections and avoid spurious tracts (ACC: *z* = 52, *y* = 50, *y* = −60; PMC: *y* = −52; SMG: *y* = −58; PCC: *z* = 42, *y* = −68; Hipp: *z* = 18, *z* = −32).

An example structural connectivity exclusion mask setup is illustrated in S12 Fig.

To control for streamline propagation, we configured each tractography analysis to propagate toward and not past the target ROI. This means for probabilistic tractography analyses, the target ROI was used as both the termination and waypoint mask. For example, when analyzing the connection from LCL to ACC, ACC was used as the termination and waypoint mask.

Structural connectivity strength was operationalized as the number of streamlines that arrived at the target from the seed region (‘waytotal’). As Probtrackx2 was set to send 10,000 streamlines from each voxel of the seed mask, the total number of streamlines sent is 10,000 multiplied by the number of voxels in the seed mask. To correct for bias from different seed volume sizes, the way total was divided by the number of voxels in the seed of origin. Then, to control for directionality within the same tractograms circuit (e.g., LCL-to-ACC and ACC-to-LCL), corrected streamline counts were averaged. This averaged corrected streamline is termed ‘connectivity strength’ and has arbitrary units (a.u.). Composite group tractograms for each circuit were created by averaging each direction of the circuit for every individual, then adding all the participant images together.

### Dynamic causal modeling

Dynamic causal modeling (DCM) [44] is a widely used technique in neuroimaging to compare different hypothetical model structures and estimate parameters describing the influence (i.e., excitatory, inhibitory, none) of one ROI on another [109]. Bayesian model comparison assigns a likelihood to each model considered. Estimated parameters are expressed in Hz and are accompanied by posterior probabilities signifying the parameter’s strength of evidence (0.0–1.0: >0.5 = “weak”, >0.75 = “positive”, >0.95 = “strong”, >0.99 = “very strong”). When extracting parameters from model comparisons, a Bayesian model average weights each model by its probability, meaning average parameter estimates are influenced more by more likely models.

DCM was performed in SPM12. Prior analyses in this study modeled working memory and autobiographical memory differently. Specifically, working memory scans were modeled with 2 regressors (“working memory” and “control” conditions) while autobiographical memory scans were modeled with 4 regressors (“autobiographical onset”, “autobiographical block”, “control onset”, and “control block”). Therefore, to facilitate comparisons across tasks in the DCM, both tasks were remodeled with 2 regressors representing “experimental” trials (“working memory” from working memory and “autobiographical” from autobiographical memory) or “control” trials. These “experimental-control” models were used to derive whole-brain experimental versus control contrast maps used in identifying circuit analysis ROIs, and to calculate experimental versus control contrast values for each ROI in Fig 3B.

ROI time series for ACC, PMC, SMG, and PCC were extracted from smoothed data, while LCL time series were extracted from unsmoothed, SRCC-corrected data. DCMs modeled both “experimental” and “control” conditions. Because images were not slice-time corrected, the model specification slice-timing parameter was set to the middle slice by specifying a time equal to the TR/2. DCMs used bilinear neural models, with one state per region, no stochastic effects, fit with time series, and without mean-centering of experimental input. This means experimental modulation parameters represent the change in effective connectivity in a projection from its value during the model’s implicit baseline, which here captured the ITI.

Because dMRI confirmed the presence of anatomical connections between LCL and all circuit analysis ROIs, all first level models included bidirectional inherent connectivity between LCL and ACC, PMC, SMG, and PCC, as well as inhibitory self-connections in all regions, which is a DCM default. “Experimental” condition-induced modulation of effective connectivity parameters and “experimental” condition driving input were modeled to test the hypothesis that the claustrum receives a network initiation signal and broadcasts it to task-associated regions. Specifically, the 4 circuit analysis ROIs yielded 16 models of claustrum output, comprising all logical combinations from experimental-induced modulation of claustrum projections to all 4 ROIs down to the null model, in which no claustrum projections were altered by task conditions. These 16 models were crossed with 5 possible sources of experimental input to the system: ACC, PMC, SMG, PCC, and LCL, with experimental input to LCL representing any other origin of task-related information such as primary sensory or other association cortices. This yielded 80 total hypothetical models.

A “full model” where all claustrum projections were modulated by experimental stimuli and all 5 ROIs received experimental driving input was estimated for all PIOP1 and PIOP2 working memory scans as well as all autobiographical memory scans [110]. All estimated first-level models were compiled into a fully connected second-level Parametric Empirical Bayes model [111]. A Bayesian model comparison was performed using the parameters of this model to assess the likelihood of all 80 hypothetical models. Bayesian model average parameters were also calculated, characterizing the task-induced change in effective connectivity of each claustrum projection by averaging the parameters across all models while weighting model parameters based on the calculated model probability. This means models deemed more likely will have a larger influence on Bayesian model average parameters than models deemed less likely.

Because comparisons of claustrum effective connectivity between memory tasks were desired, the second level design matrices contained two columns coded to specify (1) effective connectivity in task A (either working or autobiographical memory, depending on analysis) and (2) the difference in effective connectivity between task B (the other memory task, depending on analysis) and task A. DCM task comparison results are depicted in S6 (LaINS), S7 (LPulv), and S9 Figs (LCL). Predictive validity of DCM model parameters was assessed with a leave-one-out cross-validation procedure [111]. A second-level Parametric Empirical Bayes model was fitted to every first-level model, but one, and specified model parameters were used to predict task state (working memory versus autobiographical memory) in the left-out model. This process was repeated for every first-level model to assess prediction accuracy. Separate cross-validation procedures were performed for claustrum input and claustrum output parameters.

LaINS and LPulv DCMs used the same procedure except for substituting smoothed LaINS or LPulv time series for LCL time series. Analogously to task state prediction, DCM cross-validation was performed to assess seed region predictive validity. Second-level models were designed containing LCL and either LaINS or LPulv first-level models from the same task. Second-level design matrices contained two columns coded to specify (1) LCL effective connectivity and (2) the difference in effective connectivity between LCL and the other seed, LaINS or LPulv. Leave-one-out cross-validation was then performed to assess accuracy in predicting seed region separately using seed output parameters.

### Statistical analysis

Statistical details of experiments and the meaning of error bars are specified in figure legends. Whole-brain contrast maps were generated in SPM12. Results were voxel-wise thresholded at *p* < 0.001, followed by cluster correction to yield clusters significant at *p*-FWE < 0.05.

dMRI analyses were performed in SPSS 29.0.2.0. The Kolmogorov–Smirnov test, which is robust for samples >50 participants [112], was used to determine whether connectivity strengths were normally distributed. All connectivity strengths of interest had a non-normal distribution (*p* < 0.05). To compare connectivity strength with each target ROI (ACC, PMC, SMG, PCC, and Hipp) within each seed region (LCL, LaINS, and LPulv), three Related-Samples Friedman’s Two-Way ANOVA by Ranks were performed with significance set at *p* < 0.05. Dunn’s test post hoc analyses were run to determine which circuits were significantly different, with significance adjusted using Bonferroni correction for multiple comparisons. Then, five Related-Samples Friedman’s Two-Way ANOVA by Ranks were run to directly compare connectivity to the five target ROIs across seed regions with significance set to *p* < 0.05. For example, one analysis compared the LCL-ACC, LaINS-ACC, and LPulv-ACC connections. Again, Dunn’s test post hoc analyses were run with significance corrected for multiple comparisons using a Bonferroni correction. After initial Bonferroni correction, significance was set at *p* < 0.01 to account for comparisons between five ANOVAs. All *p*-values from structural connectivity analyses are recorded in S7 and S8 Tables.

DCM analyses were performed in SPM12 using SPM conventions to classify results based on posterior probability (>0.5 = “weak”, >0.75 = “positive”, >0.95 = “strong”, >0.99 = “very strong”). Significance of DCM cross-validation analyses was derived using the “Predict (cross-validation)” module in SPM12.

Significance of ROI–ROI resting state functional connectivity was calculated using paired-sample *t*-tests in MATLAB.

All other statistical analyses were performed in GraphPad Prism 9.4.0 with significance defined as *p* < 0.05. Sidak’s multiple comparisons test was used to adjust *p*-values in ANOVA post hoc comparisons in GraphPad Prism. When performing multiple statistical tests of other kinds (e.g., multiple Spearman *r* tests), multiple comparisons correction was performed using Benjamini–Hochberg FDR correction in MATLAB.

### Figures

Structural connectivity strength comparison figures were generated in GraphPad Prism v10.3.1. All other figures were generated in GraphPad Prism v9.4.0 or MRICroGL [113]. Group tractogram figures for LCL connections with network representative ROIs were thresholded at 50%, meaning fibers detected in at least 50% of participants are shown.

## Supporting information

S1 Data Excel file containing underlying, individual subject, numerical data for main manuscript figures not depicting effective connectivity parameters. Each sheet corresponds to a specific figure panel. Data and code used to produce effective connectivity figures can be found in https://osf.io/akps6.
(XLSX)

S2 Data Excel file containing underlying, individual subject, numerical data for supplemental figures not depicting effective connectivity parameters. Each sheet corresponds to a specific figure panel. Data and code used to produce effective connectivity figures can be found in https://osf.io/akps6.
(XLSX)

S1 Fig Dataset-specific whole-brain and claustrum responses during working memory. **(A)** BOLD signal increases (warm) and decreases (cool) during working memory in PIOP1 (*n* = 198). **(B)** Average parameter estimation (regression slope) of condition-related BOLD signal change detected significantly increased bilateral claustrum activation in working memory compared to control trials in PIOP1 (two-way ANOVA main effect of condition: *F* (1, 394) = 90.25, *p* < 0.0001; post hoc LCL working memory vs. control: *p* < 0.0001; post hoc RCL working memory vs. control: *p* < 0.0001). No main effect of hemisphere (*F* (1, 394) = 0.4013, *p* = 0.5268) or condition x hemisphere interaction (*F* (1, 394) = 1.787, *p* = 0.1820) were detected. **(C)** BOLD signal increases (warm) and decreases (cool) during working memory in PIOP2 (*n* = 222). **(D)** Two-way ANOVA detected significantly increased bilateral claustrum activation in working memory compared to control trials in PIOP2 (main effect of condition: *F* (1, 442) = 58.74, *p* < 0.0001; post hoc LCL working memory vs. control: *p* < 0.0001; post hoc RCL working memory vs. control: *p* < 0.0001). No main effect of hemisphere (*F* (1, 442) = 1.312, *p* = 0.2526) or condition × hemisphere interaction (*F* (1, 442) = 0.7121, *p* = 0.3992) were detected. Axial slice montages display *z* = 0, 10, 20, 30, 40, 50. Bar graphs display means with 95% confidence intervals. The data underlying bar graphs can be found in S2 Data.
(TIF)

S2 Fig Dataset-specific working memory task recruitment of resting-state networks. **(A)** Salience (red), fronto-parietal (purple), dorsal attention (orange), and default mode (blue) networks from PIOP1 resting state group-ICA (top) with PIOP1 working memory BOLD increases (warm, middle) and decreases (cool, bottom) overlaid. **(B)** Same for PIOP2. Axial slice montages display *z* = 0, 10, 20, 30, 40, 50.
(TIF)

S3 Fig “Match” and “different” working memory trials elicit bilateral network and claustrum responses. **(A)** BOLD signal increases (warm) and decreases (cool) from the “match - control” contrast in PIOP1, PIOP2, and the combined sample with accompanying LCL and RCL “match - control” activation (PIOP1: LCL *t* = 5.745, *p*-FDR < 0.0001; RCL *t* = 5.312, *p*-FDR < 0.0001; PIOP2: LCL *t* = 4.472, *p*-FDR < 0.0001; RCL *t* = 3.754, *p*-FDR = 0.0002; Combined: LCL *t* = 7.251, *p*-FDR < 0.0001; RCL *t* = 6.447, *p*-FDR < 0.0001). **(B)** BOLD signal increases (warm) and decreases (cool) from the “different - control” contrast in PIOP1, PIOP2, and the combined sample with accompanying LCL and RCL “different - control” activation (PIOP1: LCL *t* = 7.898, *p*-FDR < 0.0001; RCL *t* = 6.172, *p*-FDR < 0.0001; PIOP2: LCL *t* = 6.439, *p*-FDR < 0.0001; RCL *t* = 5.653, *p*-FDR < 0.0001; Combined: LCL *t* = 10.13, *p*-FDR < 0.0001; RCL *t* = 8.367, *p*-FDR < 0.0001). Axial slice montages display *z* = 0, 10, 20, 30, 40, 50. Bar graphs display means with 95% confidence intervals. The data underlying bar graphs can be found in S2 Data.
(TIF)

S4 Fig Dataset-specific whole-brain and claustrum responses during different vs. match working memory trials. **(A)** BOLD signal increases (red) and decreases (blue) from the “different - match” contrast in PIOP1. **(B)** LCL and RCL “different - match” activation in PIOP1 (LCL *t* = 3.532, *p*-FDR = 0.0016; RCL *t* = 1.447, *p*-FDR = 0.1496). **(C)** BOLD signal increases (red) and decreases (blue) from the “different - match” contrast in PIOP2. **(D)** LCL and RCL “different - match” activation in PIOP2 (LCL *t* = 3.287, *p*-FDR = 0.0016; RCL *t* = 3.274, *p*-FDR = 0.0016). Bar graphs display means with 95% confidence intervals. The data underlying bar graphs can be found in S2 Data.
(TIF)

S5 Fig Dataset-specific whole-brain and claustrum responses during correct vs. incorrect working memory trials. **(A)** No BOLD signal increases or decreases were observed in the “correct - incorrect” contrast in PIOP1 (*n* = 195). **(B)** LCL and RCL “correct - incorrect” activation in PIOP1 (LCL *W* = 2,906, *p* = 0.0656; RCL *W* = 2,138, *p* = 0.1761). **(C)** No BOLD signal increases or decreases were observed in the “correct - incorrect” contrast in PIOP2 (*n* = 221). **(B)** LCL and RCL “correct - incorrect” activation in PIOP2 (LCL *W* = 1,287, *p* = 0.4992; RCL *W* = 711, *p* = 0.7091). Bar graphs display means with 95% confidence intervals. The data underlying bar graphs can be found in S2 Data.
(TIF)

S6 Fig Left anterior insula task response, effective connectivity, and structural connectivity profiles. **(A) Top:** LaINS in salience network (*z* = −5). **Middle:** “Experimental - control” contrasts revealed significant LaINS activation in WM and Autobio (WM: *t* = 10.17, *p*-FDR < 0.0001; Autobio: *t* = 4.95; *p*-FDR < 0.0001). **Bottom:** Hybrid event-block modeling of Autobio detected significantly increased LaINS activation during autobiographical memory and control trials (two-way ANOVA main effect of condition: *F* (1, 68) = 18.08, *p* < 0.0001; main effect of time point: *F* (1, 68) = 54.64, *p* < 0.0001; condition x time point interaction: *F* (1, 68) = 7.599, *p* = 0.0075; post hoc Autobio onset vs. Autobio block: *p* < 0.0001; post hoc control onset vs. control block: *p* = 0.0033; post hoc Autobio onset vs. control onset: *p* < 0.0001). Bar graphs display means with 95% confidence intervals. **(B) Top:** Estimated DCM output parameters during WM and differences with Autobio. **Bottom:** Estimated DCM output parameters during Autobio and differences with WM. 95% confidence intervals and posterior probabilities signifying evidence strength (> 0.5 = “weak”, > 0.75 = “positive”, > 0.95 = “strong”, > 0.99 = “very strong”) accompany bars. **(C)** LaINs exhibited preferential structural connectivity with PMC. Select comparisons shown for clarity. Plot displays medians with 95% confidence intervals. The data and code used to produce this figure can be found in S2 Data and https://osf.io/akps6.
(TIF)

S7 Fig Left pulvinar task response, effective connectivity, and structural connectivity profiles. **(A) Top:** LPulv in left thalamus (Cole and colleagues, 2019; *z* = 8). **Middle:** “Experimental - control” contrasts revealed significant LPulv activation in WM and Autobio tasks (WM: *t* = 6.47, *p*-FDR < 0.0001; Autobio: *t* = 2.57; *p*-FDR = 0.0149). **Bottom:** Hybrid event-block modeling of Autobio detected significantly increased LPulv activation at autobiographical memory trial onset (two-way ANOVA main effect of condition: *F* (1, 68) = 13.25, *p* < 0.0005; main effect of time point: *F* (1, 68) = 4.640, *p* < 0.0348; condition x time point interaction: *F* (1, 68) = 9.595, *p* = 0.0028; post hoc Autobio onset vs. Autobio block: *p* = 0.0008; post hoc Autobio onset vs. control onset: *p* < 0.0001). However, Autobio onset activation was positively correlated with subject motion (S6 Table). Bar graphs display means with 95% confidence intervals. **(B) Top:** Estimated DCM output parameters during WM and differences with Autobio. **Bottom:** Estimated DCM output parameters during Autobio and differences with WM. 95% confidence intervals and posterior probabilities signifying evidence strength (>0.5 = “weak”, >0.75 = “positive”, >0.95 = “strong”, >0.99 = “very strong”) accompany bars. **(C)** LPulv exhibited preferential structural connectivity with Hipp. Select comparisons shown for clarity. Plot displays medians with 95% confidence intervals. The data and code used to produce this figure can be found in S2 Data and https://osf.io/akps6.
(TIF)

S8 Fig Larger, longer, and less specific LaINS than LCL responses to autobiographical memory scan stimuli. One sample *t*-tests of LaINS and LCL BOLD responses to autobiographical memory scan stimuli detected significant activation above baseline in LaINS to all conditions, but only to Autobio onset in LCL (LaINS: Autobio onset *t* = 8.974, *p*-FDR < 0.0001; Autobio block *t* = 4.907, *p*-FDR < 0.0001; Control onset *t* = 4.455, *p*-FDR = 0.0002; Control block *t* = 2.806, *p*-FDR = 0.0132; LCL: Autobio onset *t* = 6.194, *p*-FDR < 0.0001; Autobio block *t* = 0.6129, *p*-FDR = 0.5440; Control onset *t* = 1.936, *p*-FDR = 0.0817; Control block *t* = 1.249, *p*-FDR = 0.2516). Two-way ANOVA detected significantly greater LaINS than LCL signal (main effect of ROI: *F* (1, 68) = 40.71, *p* < 0.0001; main effect of condition: *F* (2.086, 141.9) = 43.46, *p* < 0.0001; ROI x condition interaction: *F* (3, 204) = 10.20, *p* < 0.0001), with post hoc comparisons identifying greater LaINS than LCL signal during Autobio onset (*p* < 0.0001), Autobio block (*p* = 0.0008), and Control onset (*p* = 0.0104), but not Control block (*p* = 0.3049). Bar graph displays means with 95% confidence intervals. The data underlying this figure can be found in S2 Data.
(TIF)

S9 Fig Network representative region ROIs overlaid on isolated resting-state networks from combined PIOP1 and PIOP2 group-ICA. Network representative region ROIs (black circles) overlaid on cognitive control networks for better visualization. **(A)** ACC in salience network. **(B)** PMC in fronto-parietal network. **(C)** SMG in dorsal attention network. **(D)** PCC in default mode network.
(TIF)

S10 Fig Left claustrum DCM task comparisons. **(A) Left:** Estimated DCM input parameters during WM and differences with Autobio. **Right:** Estimated DCM input parameters during Autobio and differences with WM. **(B) Left:** Estimated DCM output parameters during WM and differences with Autobio. **Right:** Estimated DCM output parameters during Autobio and differences with WM. 95% confidence intervals and posterior probabilities signifying evidence strength (>0.5 = “weak”, >0.75 = “positive”, >0.95 = “strong”, >0.99 = “very strong”) accompany bars. The data and code used to produce this figure can be found in https://osf.io/akps6.
(TIF)

S11 Fig Claustrum results were not materially impacted by removal of the first 10 volumes. **(A)** LCL and RCL activation during autobiographical memory scans, reproduced from Fig 2C. **(B)** LCL and RCL activation during autobiographical memory scans following preprocessing which included removal of the initial 10 volumes. Bilateral claustrum results are qualitatively similar (2-way ANOVA main effect of condition: *F* (2.149, 146.2) = 13.69, *p* < 0.0001; main effect of hemisphere: *F* (1, 68) = 8.080, *p* = 0.0059; condition x hemisphere interaction: *F* (3, 204) = 3.559, *p* = 0.0152; post hoc LCL Autobio onset versus Autobio block: *p* < 0.0001; post hoc LCL Autobio onset vs. control onset: *p* = 0.0056). Bar graphs display means with 95% confidence intervals. The data underlying this figure can be found in S2 Data.
(TIF)

S12 Fig Representative dMRI analysis exclusion masks. Representative structural connectivity analysis exclusion mask (yellow) setup for the connection between LCL (black, top middle) and PCC (blue, top left). Analyses used combinations of unanalyzed target ROIs, nearby notable ROIs, and additional planes for specificity. LCL-PCC analyses used ROI exclusion masks for ACC (top left), PMC (top middle, bottom left), SMG, and Hipp, as well as masks for thalamus, insular cortex, and amygdala. Exclusion planes for LCL-PCC included a mid-sagittal plane (*x* = 0), an axial plane at *z* = 42, and a coronal plane at *y* = −68. Images displayed in radiological orientation.
(TIF)

S1 Table High likelihood of misattribution in working memory GLMs with individual task component regressors. Variance Inflation Factors (VIFs) reveal high likelihood of misattribution when modeling each task component (alert, encoding, retention, test, response) of working memory and control trials. Common “rules of thumb” regard VIFs exceeding 5 or 10 as suggesting high levels of multicollinearity that can severely impact model stability [84].
(PDF)

S2 Table No significant positive correlations between subject motion and claustrum BOLD signal change. Spearman *r* tests (average framewise displacement [FD] values were not normally distributed) find no significant correlations between individual participant LCL or RCL BOLD signal activation parameters and average motion during task scans. Working memory correlations used the working memory condition vs. implicit baseline contrast, and autobiographical memory correlations used the autobiographical memory onset condition vs. implicit baseline contrast. Implicit baseline in both models encompassed inter-trial intervals (ITIs) and volumes obtained prior to and following task runs. Only RCL-AVG FD during PIOP1 working memory yielded an uncorrected *p*-value less than 0.05 (*r* = −0.1578; *p* < 0.0264), but the inverse correlation revealed RCL signal decreased with greater motion, meaning motion-induced noise potentially biased observations away from significant condition effects.
(PDF)

S3 Table Working memory task accuracy by trial type. Working memory trials with responses were classified as correct or incorrect. If no response was made in the 1 s response phase, the trial was classified as a timeout. Control trials were only classified depending on if a response was made (“hit”) or not (“miss”) regardless of button press accuracy. Accuracy (defined as percent correct) was compared between working memory “different” and “match” trials. Because accuracy percentages were not normally distributed, Wilcoxon matched-pairs signed rank tests were performed to compare accuracies between conditions. “Different” and “match” accuracies were not significantly different in either dataset (PIOP1: *p* = 0.0797; PIOP2: *p* = 0.1815).
(PDF)

S4 Table Working memory task mean reaction time by trial type. Mixed Effects Analysis did not detect a significant effect of condition (different vs. match) or accuracy (correct vs. incorrect) on mean reaction time in either PIOP1 or PIOP2. Only a condition x accuracy interaction was detected in PIOP1 (*p* = 0.0132), where incorrect responses were made faster than correct responses in “different” trials.
(PDF)

S5 Table No risk of misattribution in autobiographical memory GLMs when using hybrid event-block format. All VIFs using hybrid event-block format in autobiographical memory task GLMs are less than 2, reflecting no risk of model instability or misattribution.
(PDF)

S6 Table Correlations between subject motion and LaINS and LPulv BOLD signal change. Spearman *r* tests (average framewise displacement [FD] values were not normally distributed) find no significant correlations between individual participant LaINS BOLD signal activation and average motion during task scans. However, a significant positive correlation was found between LPulv BOLD signal activation and average motion during autobiographical memory scans (*r* = 0.5280, *p*-FDR = 0.0066), calling into question the validity of the observed task-induced BOLD increase in the region. Working memory correlations used the working memory condition vs. implicit baseline contrast, and autobiographical memory correlations used the autobiographical memory onset condition versus implicit baseline contrast. Implicit baseline in both models encompassed inter-trial intervals (ITIs) and volumes obtained prior to and following task runs.
(PDF)

S7 Table Within-seed region structural connectivity post hoc comparison *p*-values. For each seed region (LCL, LaINS, LPulv) structural connectivity with that region was compared between all pairs of network representative regions in the combined AOMIC PIOP1&2 dataset. For example, the first row compares structural connectivity strength between LCL-PCC and LCL-Hipp connections, with LCL-PCC exhibiting significantly greater structural connectivity. Rows are arranged so the two strongest connections for any seed are compared in that seed’s bottom row. All *p*-values are adjusted using Bonferroni correction within each seed analysis.
(PDF)

S8 Table Between-seed ROI structural connectivity post hoc comparison *p*-values. For each network region, structural connectivity with that region was compared between all pairs of seed regions in the combined AOMIC PIOP1&2 dataset. For example, the first row compares structural connectivity strength between LPulv-ACC and LaINS-ACC connections, with LPulv-ACC exhibiting significantly stronger structural connectivity. Rows are arranged so the two strongest connections for any network region are compared in that region’s bottom row. All *p*-values are adjusted using Bonferroni correction within each region analysis. Because comparisons include 5 separate ANOVAs, significance is set at *p* < 0.05/5, or *p* < 0.01.
(PDF)

S9 Table Left claustrum resting state functional connectivity with networks and network representative regions. Left claustrum exhibited statistically significant ROI-ROI functional connectivity at rest in the combined AOMIC PIOP1&2 dataset with all selected network representative ROIs and with all identified cognitive control networks. Significance of ROI-ROI resting state functional connectivity was calculated using paired-sample t-tests in MATLAB.
(PDF)

S10 Table Dynamic causal modeling cross-validation. Leave-one-out cross-validation was performed to assess model effectiveness in predicting task state (working memory using combined PIOP1&2 datasets vs. autobiographical memory) and seed region. Pearson’s correlation coefficients, uncorrected *p*-values, and *p* values after Benjamini–Hochberg False Discovery Rate correction accounting for all statistical tests in the table are listed. Note the similarity in results when using “input” parameters across all seed regions. Seed region output parameters were statistically significant in distinguishing LCL vs. LaINS during working memory, with a trend toward significance in the smaller autobiographical memory dataset.
(PDF)

S11 Table Input Bayesian model comparison probabilities. Probabilities assigned to sources of experimental input to seed regions during working and autobiographical memory tasks. In all models the seed region was used as the fifth possible source of input, modeling all other possible sources of experimental input. Note that input source probabilities are identical across seed regions.
(PDF)

S12 Table Output Bayesian model comparison probabilities. Probabilities assigned to models with different combinations of task-modulation of network representative region outputs. Values reveal which model structure is favored, but they do not provide insight into the parameters (e.g., excitatory or inhibitory) describing the models.
(PDF)

## Abbreviations

ACC: anterior cingulate cortex
AMI: Autobiographical Memory Interview
ANOVA: analysis of variance
AOMIC: amsterdam open MRI collection
Autobio: autobiographical memory
BOLD: blood oxygenation level dependent
CSF: cerebrospinal fluid
DAN: dorsal attention network
DCM: dynamic causal models
DMN: default mode network
dMRI: diffusion MRI
EPI: echo-planar imaging
FD: framewise displacement
FDR: false discovery rate
FOV: field of view
FPN: fronto-parietal network
FWE: family-wise error
FWHM: full-width at half maximum
GLMs: general linear models
HIPP: hippocampus
HRF: hemodynamic response function
ICA: Independent Component Analysis
ITI: inter-trial interval
LaINS: Left anterior insula
LCL: left claustrum
LPulv: Left pulvinar
MNI: Montreal Neurological Institute
PCC: posterior cingulate cortex/precuneus
PMC: premotor cortex
RCL: right claustrum
ROIs: regions of interest
SMG: supramarginal gyrus
SN: salience network
SRCC: Small Region Confound Correction
TE: echo time
TR: repetition time
WM: working memory
