# Supplementary figures and images for: The human claustrum supports cognitive networks for externally and internally driven task demands
Source: PLoS Biol. 2026 Jun 26;24(6):e3003843. doi: 10.1371/journal.pbio.3003843 (PMC13308805; doi:10.1371/journal.pbio.3003843)

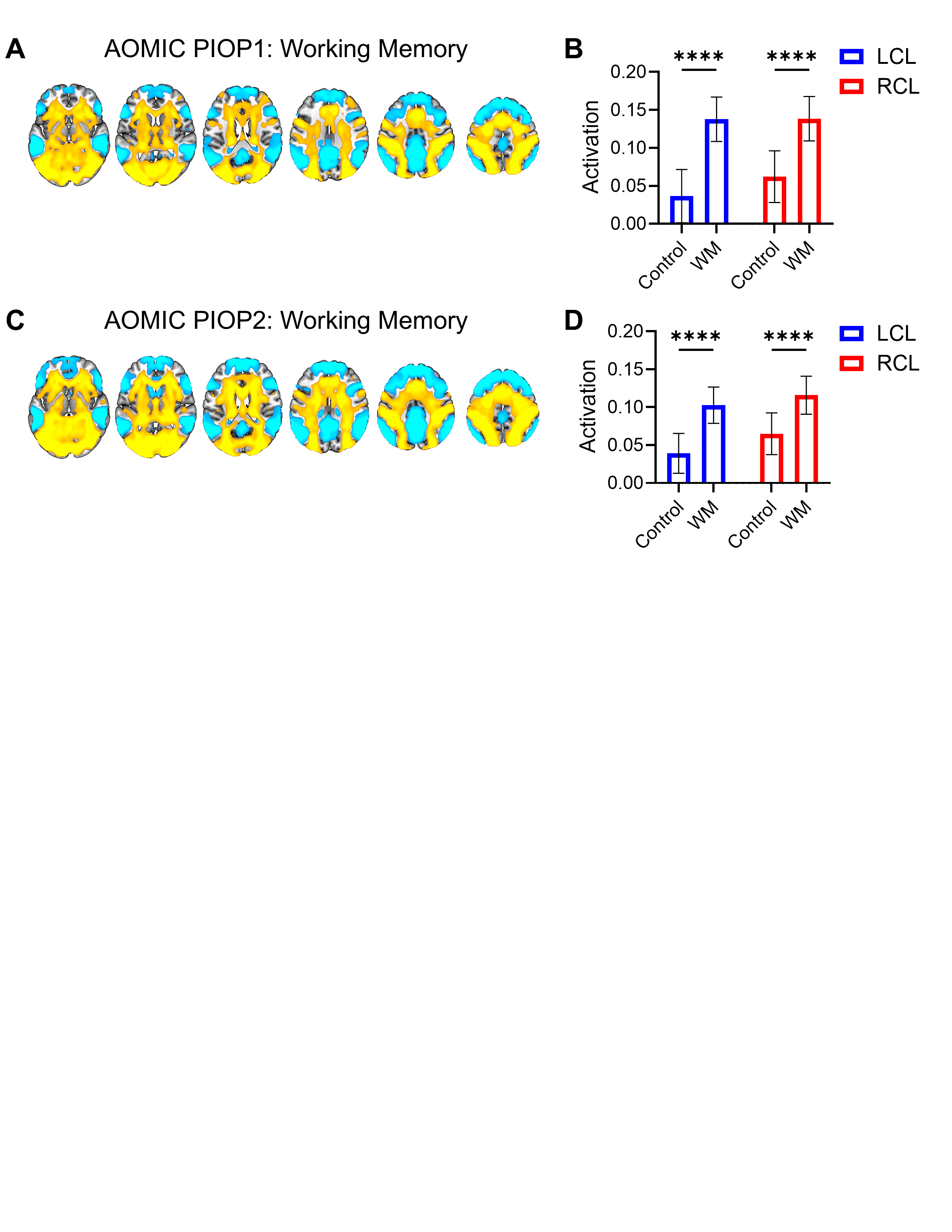

Supplement: S1 Fig — (A) BOLD signal increases (warm) and decreases (cool) during working memory in PIOP1 (n = 198). (B) Average parameter estimation (regression slope) of condition-related BOLD signal change detected significantly increased bilateral claustrum activation in working memory compared to control trials in PIOP1 (two-way ANOVA main effect of condition: F (1, 394) = 90.25, p < 0.0001; post hoc LCL working memory vs. control: p < 0.0001; post hoc RCL working memory vs. control: p < 0.0001). No main effect of hemisphere (F (1, 394) = 0.4013, p = 0.5268) or condition x hemisphere interaction (F (1, 394) = 1.787, p = 0.1820) were detected. (C) BOLD signal increases (warm) and decreases (cool) during working memory in PIOP2 (n = 222). (D) Two-way ANOVA detected significantly increased bilateral claustrum activation in working memory compared to control trials in PIOP2 (main effect of condition: F (1, 442) = 58.74, p < 0.0001; post hoc LCL working memory vs. control: p < 0.0001; post hoc RCL working memory vs. control: p < 0.0001). No main effect of hemisphere (F (1, 442) = 1.312, p = 0.2526) or condition × hemisphere interaction (F (1, 442) = 0.7121, p = 0.3992) were detected. Axial slice montages display z = 0, 10, 20, 30, 40, 50. Bar graphs display means with 95% confidence intervals. The data underlying bar graphs can be found in S2 Data. (TIF) [file pbio.3003843.s003.TIF]

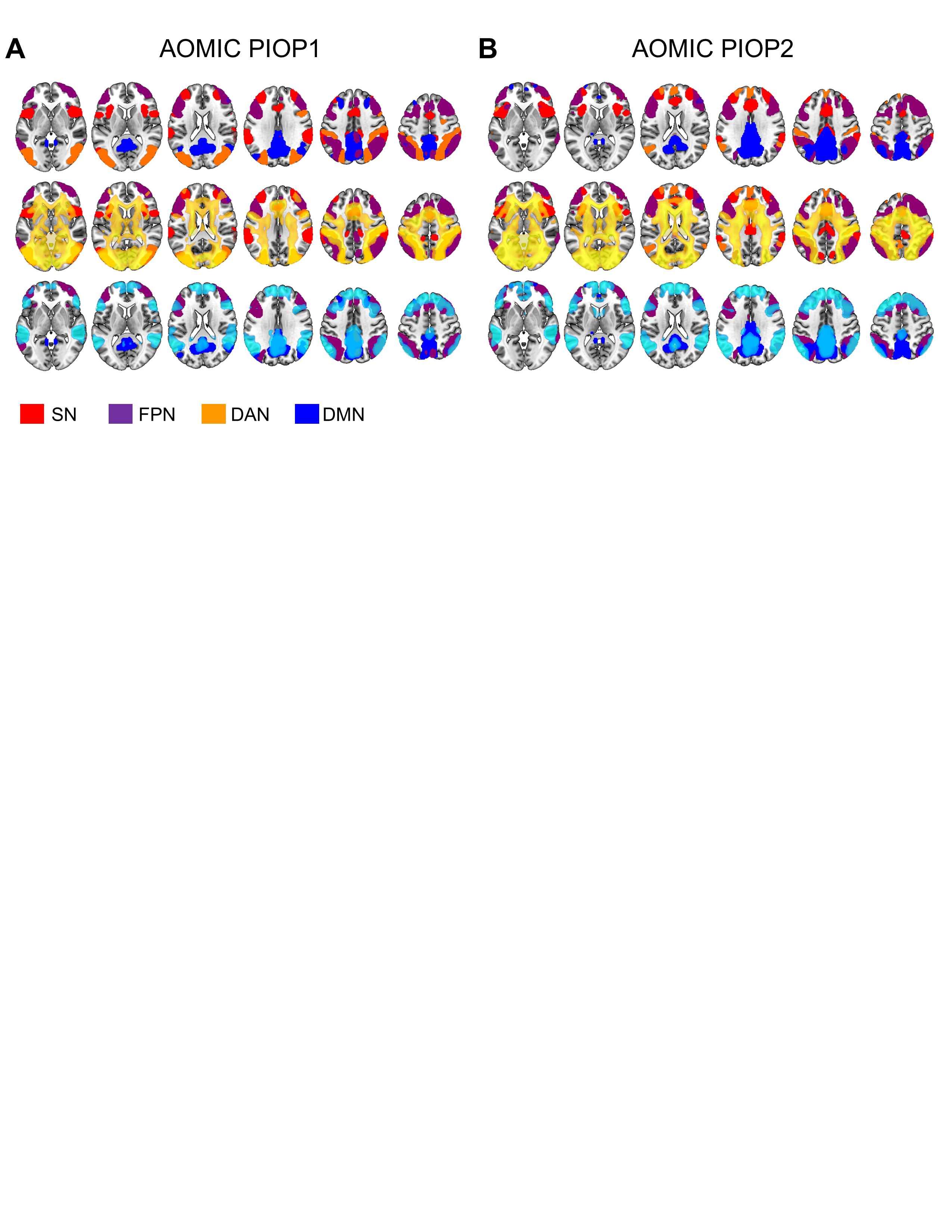

Supplement: S2 Fig — (A) Salience (red), fronto-parietal (purple), dorsal attention (orange), and default mode (blue) networks from PIOP1 resting state group-ICA (top) with PIOP1 working memory BOLD increases (warm, middle) and decreases (cool, bottom) overlaid. (B) Same for PIOP2. Axial slice montages display z = 0, 10, 20, 30, 40, 50. (TIF) [file pbio.3003843.s004.TIF]

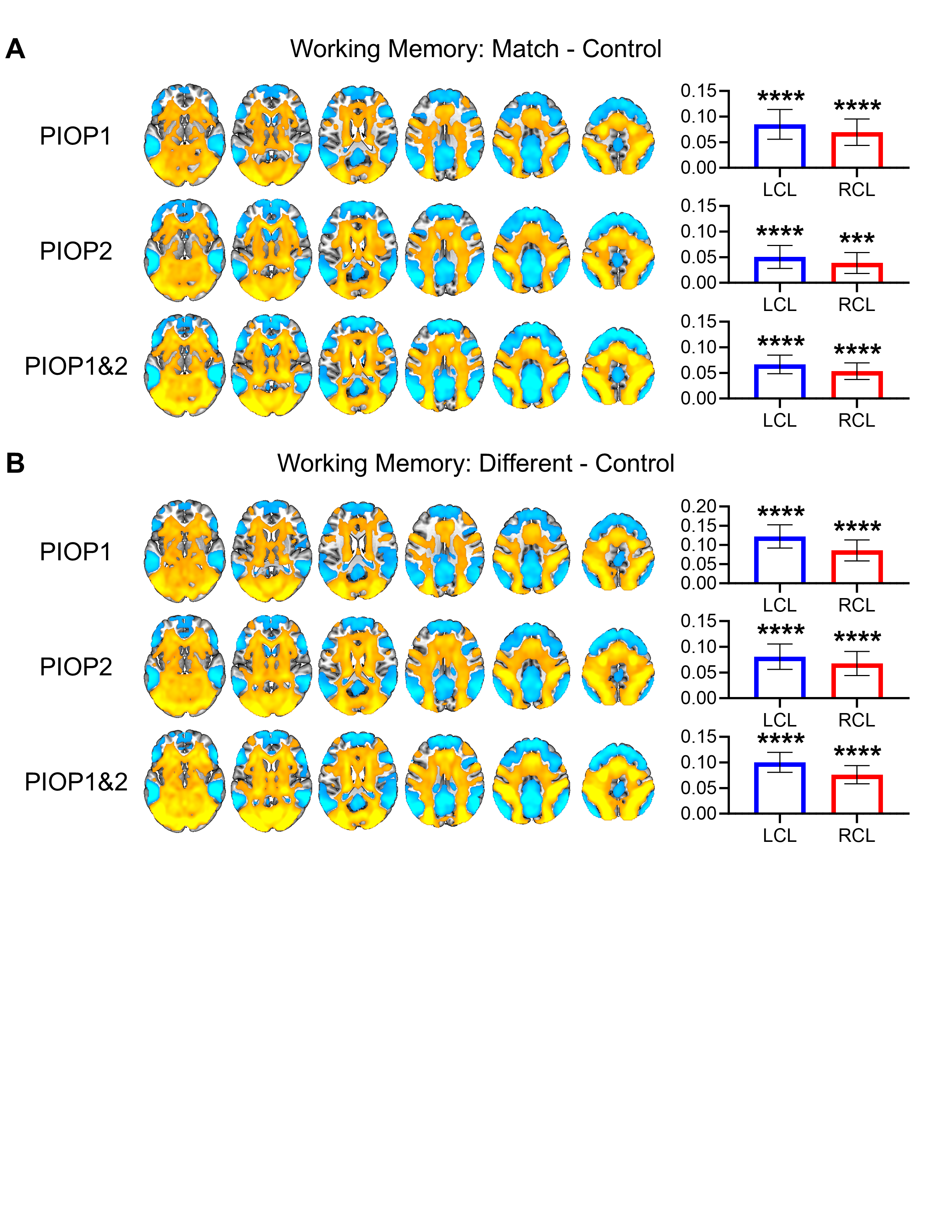

Supplement: S3 Fig — (A) BOLD signal increases (warm) and decreases (cool) from the “match - control” contrast in PIOP1, PIOP2, and the combined sample with accompanying LCL and RCL “match - control” activation (PIOP1: LCL t = 5.745, p-FDR < 0.0001; RCL t = 5.312, p-FDR < 0.0001; PIOP2: LCL t = 4.472, p-FDR < 0.0001; RCL t = 3.754, p-FDR = 0.0002; Combined: LCL t = 7.251, p-FDR < 0.0001; RCL t = 6.447, p-FDR < 0.0001). (B) BOLD signal increases (warm) and decreases (cool) from the “different - control” contrast in PIOP1, PIOP2, and the combined sample with accompanying LCL and RCL “different - control” activation (PIOP1: LCL t = 7.898, p-FDR < 0.0001; RCL t = 6.172, p-FDR < 0.0001; PIOP2: LCL t = 6.439, p-FDR < 0.0001; RCL t = 5.653, p-FDR < 0.0001; Combined: LCL t = 10.13, p-FDR < 0.0001; RCL t = 8.367, p-FDR < 0.0001). Axial slice montages display z = 0, 10, 20, 30, 40, 50. Bar graphs display means with 95% confidence intervals. The data underlying bar graphs can be found in S2 Data. (TIF) [file pbio.3003843.s005.TIF]

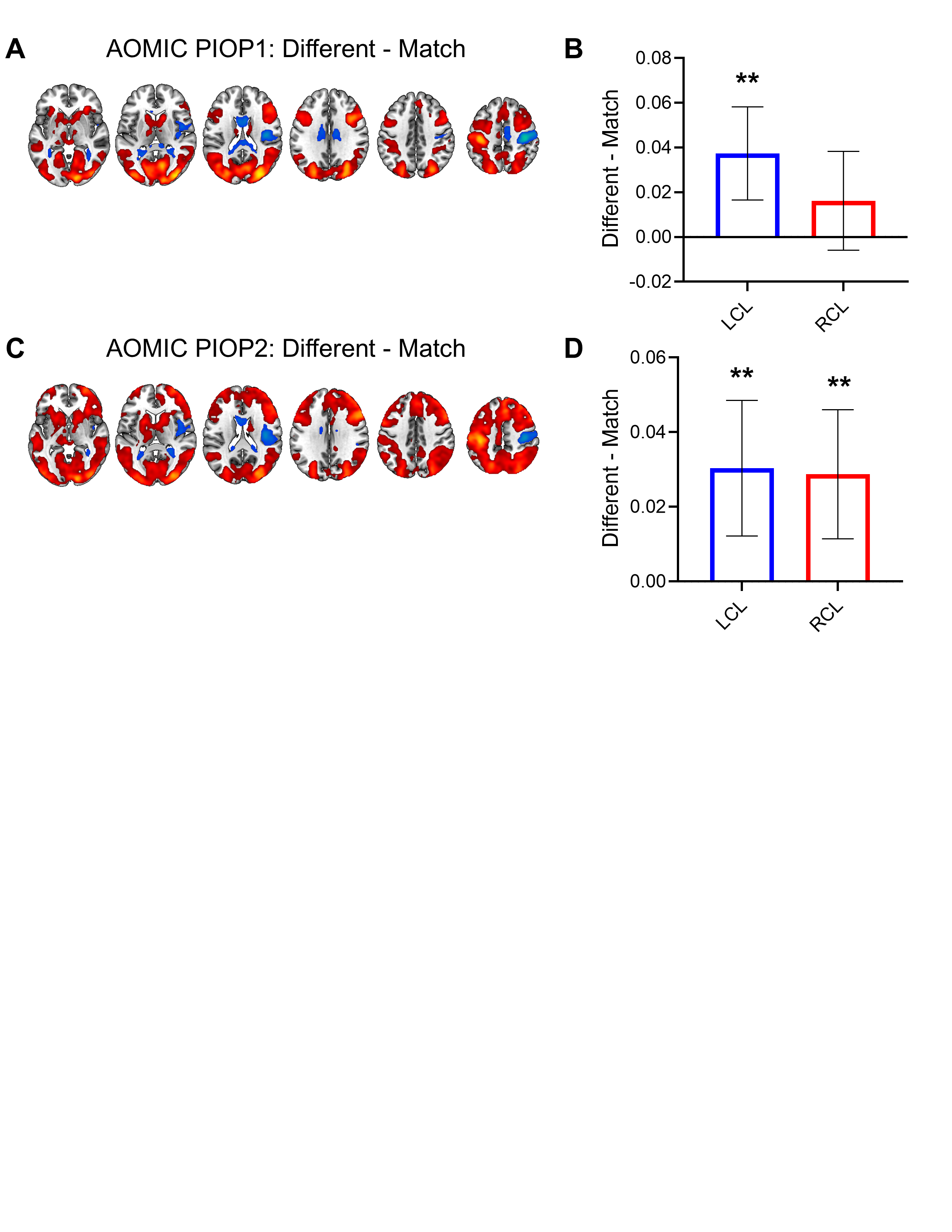

Supplement: S4 Fig — (A) BOLD signal increases (red) and decreases (blue) from the “different - match” contrast in PIOP1. (B) LCL and RCL “different - match” activation in PIOP1 (LCL t = 3.532, p-FDR = 0.0016; RCL t = 1.447, p-FDR = 0.1496). (C) BOLD signal increases (red) and decreases (blue) from the “different - match” contrast in PIOP2. (D) LCL and RCL “different - match” activation in PIOP2 (LCL t = 3.287, p-FDR = 0.0016; RCL t = 3.274, p-FDR = 0.0016). Bar graphs display means with 95% confidence intervals. The data underlying bar graphs can be found in S2 Data. (TIF) [file pbio.3003843.s006.TIF]

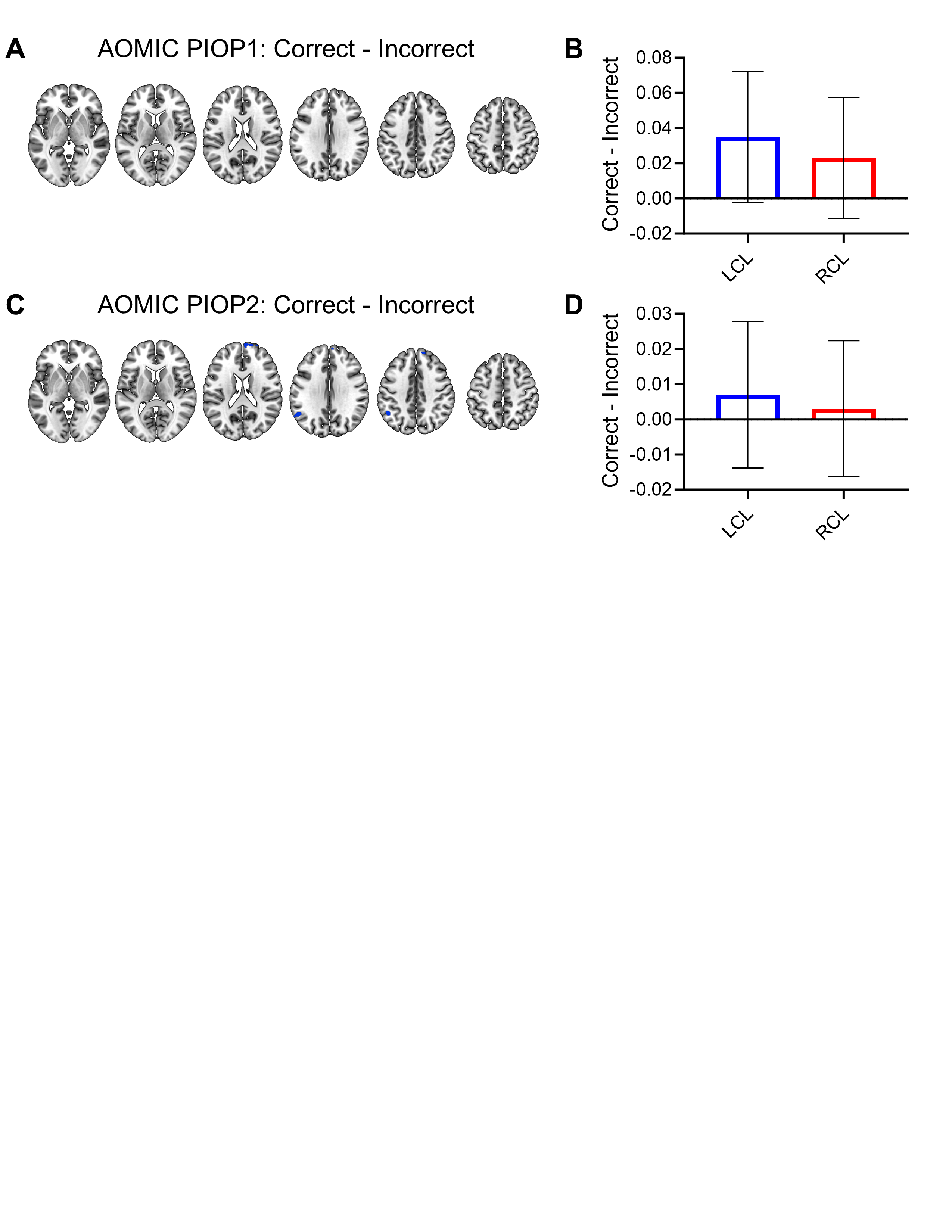

Supplement: S5 Fig — (A) No BOLD signal increases or decreases were observed in the “correct - incorrect” contrast in PIOP1 (n = 195). (B) LCL and RCL “correct - incorrect” activation in PIOP1 (LCL W = 2,906, p = 0.0656; RCL W = 2,138, p = 0.1761). (C) No BOLD signal increases or decreases were observed in the “correct - incorrect” contrast in PIOP2 (n = 221). (B) LCL and RCL “correct - incorrect” activation in PIOP2 (LCL W = 1,287, p = 0.4992; RCL W = 711, p = 0.7091). Bar graphs display means with 95% confidence intervals. The data underlying bar graphs can be found in S2 Data. (TIF) [file pbio.3003843.s007.TIF]

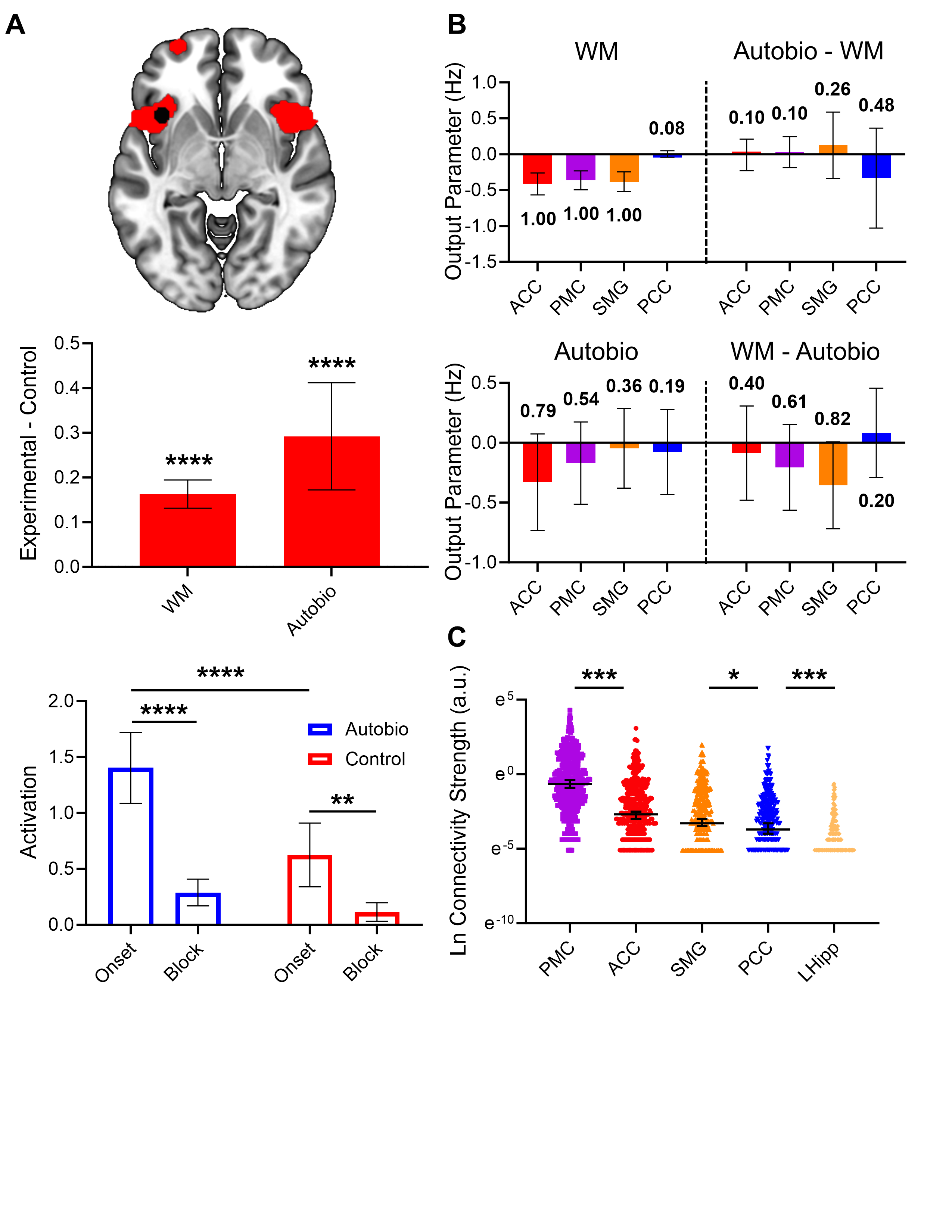

Supplement: S6 Fig — (A) Top: LaINS in salience network (z = −5). Middle: “Experimental - control” contrasts revealed significant LaINS activation in WM and Autobio (WM: t = 10.17, p-FDR < 0.0001; Autobio: t = 4.95; p-FDR < 0.0001). Bottom: Hybrid event-block modeling of Autobio detected significantly increased LaINS activation during autobiographical memory and control trials (two-way ANOVA main effect of condition: F (1, 68) = 18.08, p < 0.0001; main effect of time point: F (1, 68) = 54.64, p < 0.0001; condition x time point interaction: F (1, 68) = 7.599, p = 0.0075; post hoc Autobio onset vs. Autobio block: p < 0.0001; post hoc control onset vs. control block: p = 0.0033; post hoc Autobio onset vs. control onset: p < 0.0001). Bar graphs display means with 95% confidence intervals. (B) Top: Estimated DCM output parameters during WM and differences with Autobio. Bottom: Estimated DCM output parameters during Autobio and differences with WM. 95% confidence intervals and posterior probabilities signifying evidence strength (> 0.5 = “weak”, > 0.75 = “positive”, > 0.95 = “strong”, > 0.99 = “very strong”) accompany bars. (C) LaINs exhibited preferential structural connectivity with PMC. Select comparisons shown for clarity. Plot displays medians with 95% confidence intervals. The data and code used to produce this figure can be found in S2 Data and https://osf.io/akps6. (TIF) [file pbio.3003843.s008.TIF]

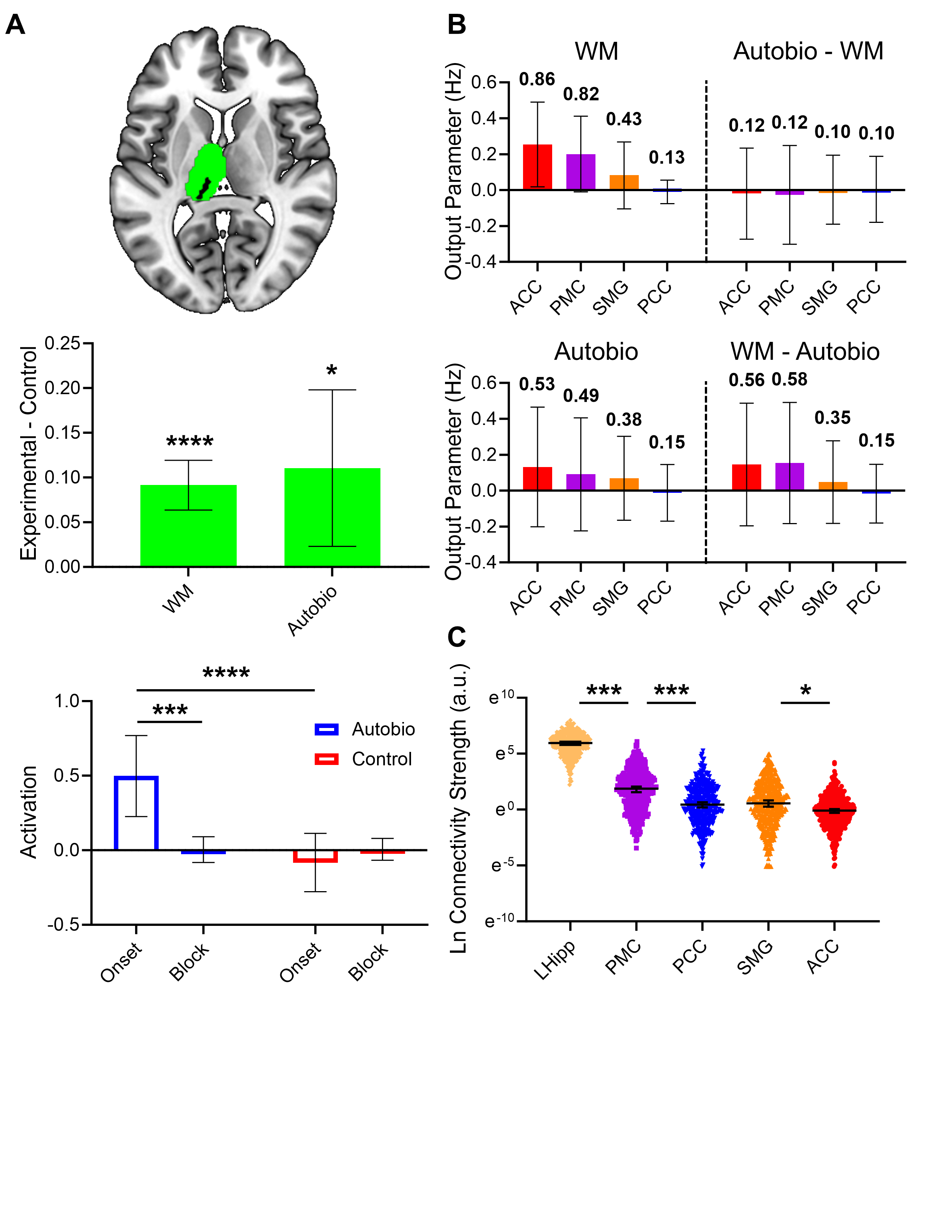

Supplement: S7 Fig — (A) Top: LPulv in left thalamus (Cole and colleagues, 2019; z = 8). Middle: “Experimental - control” contrasts revealed significant LPulv activation in WM and Autobio tasks (WM: t = 6.47, p-FDR < 0.0001; Autobio: t = 2.57; p-FDR = 0.0149). Bottom: Hybrid event-block modeling of Autobio detected significantly increased LPulv activation at autobiographical memory trial onset (two-way ANOVA main effect of condition: F (1, 68) = 13.25, p < 0.0005; main effect of time point: F (1, 68) = 4.640, p < 0.0348; condition x time point interaction: F (1, 68) = 9.595, p = 0.0028; post hoc Autobio onset vs. Autobio block: p = 0.0008; post hoc Autobio onset vs. control onset: p < 0.0001). However, Autobio onset activation was positively correlated with subject motion (S6 Table). Bar graphs display means with 95% confidence intervals. (B) Top: Estimated DCM output parameters during WM and differences with Autobio. Bottom: Estimated DCM output parameters during Autobio and differences with WM. 95% confidence intervals and posterior probabilities signifying evidence strength (>0.5 = “weak”, >0.75 = “positive”, >0.95 = “strong”, >0.99 = “very strong”) accompany bars. (C) LPulv exhibited preferential structural connectivity with Hipp. Select comparisons shown for clarity. Plot displays medians with 95% confidence intervals. The data and code used to produce this figure can be found in S2 Data and https://osf.io/akps6. (TIF) [file pbio.3003843.s009.TIF]

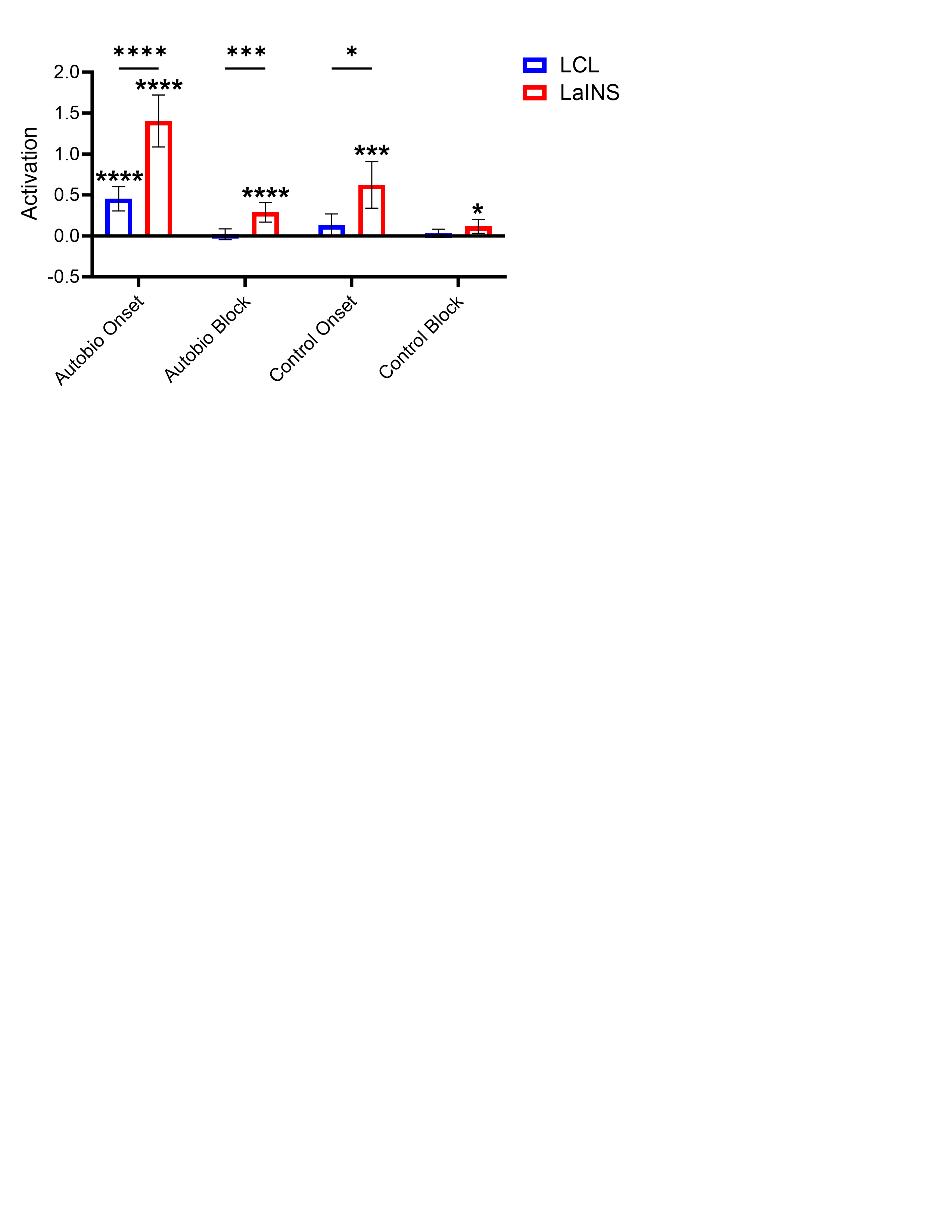

Supplement: S8 Fig — One sample t-tests of LaINS and LCL BOLD responses to autobiographical memory scan stimuli detected significant activation above baseline in LaINS to all conditions, but only to Autobio onset in LCL (LaINS: Autobio onset t = 8.974, p-FDR < 0.0001; Autobio block t = 4.907, p-FDR < 0.0001; Control onset t = 4.455, p-FDR = 0.0002; Control block t = 2.806, p-FDR = 0.0132; LCL: Autobio onset t = 6.194, p-FDR < 0.0001; Autobio block t = 0.6129, p-FDR = 0.5440; Control onset t = 1.936, p-FDR = 0.0817; Control block t = 1.249, p-FDR = 0.2516). Two-way ANOVA detected significantly greater LaINS than LCL signal (main effect of ROI: F (1, 68) = 40.71, p < 0.0001; main effect of condition: F (2.086, 141.9) = 43.46, p < 0.0001; ROI x condition interaction: F (3, 204) = 10.20, p < 0.0001), with post hoc comparisons identifying greater LaINS than LCL signal during Autobio onset (p < 0.0001), Autobio block (p = 0.0008), and Control onset (p = 0.0104), but not Control block (p = 0.3049). Bar graph displays means with 95% confidence intervals. The data underlying this figure can be found in S2 Data. (TIF) [file pbio.3003843.s010.TIF]

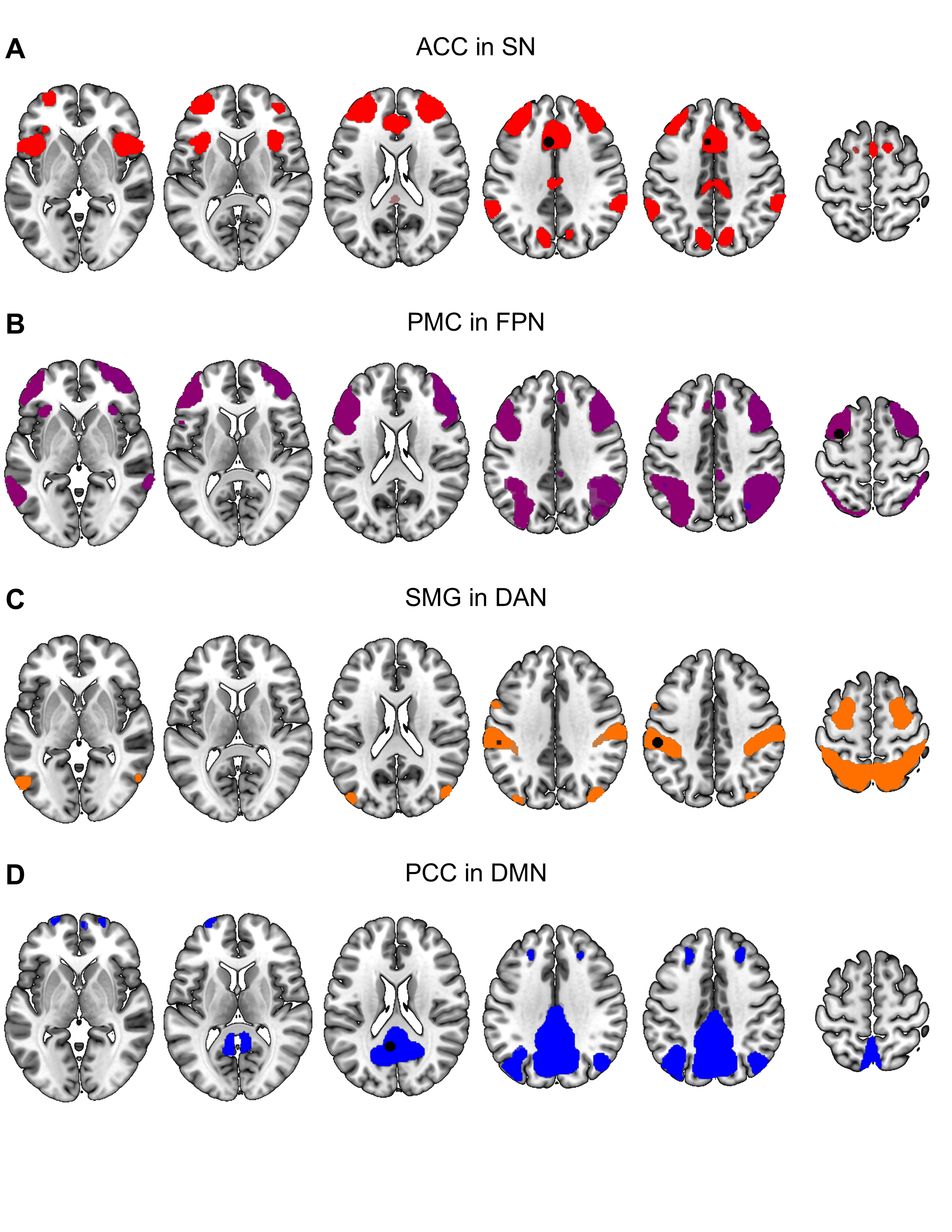

Supplement: S9 Fig — Network representative region ROIs (black circles) overlaid on cognitive control networks for better visualization. (A) ACC in salience network. (B) PMC in fronto-parietal network. (C) SMG in dorsal attention network. (D) PCC in default mode network. (TIF) [file pbio.3003843.s011.TIF]

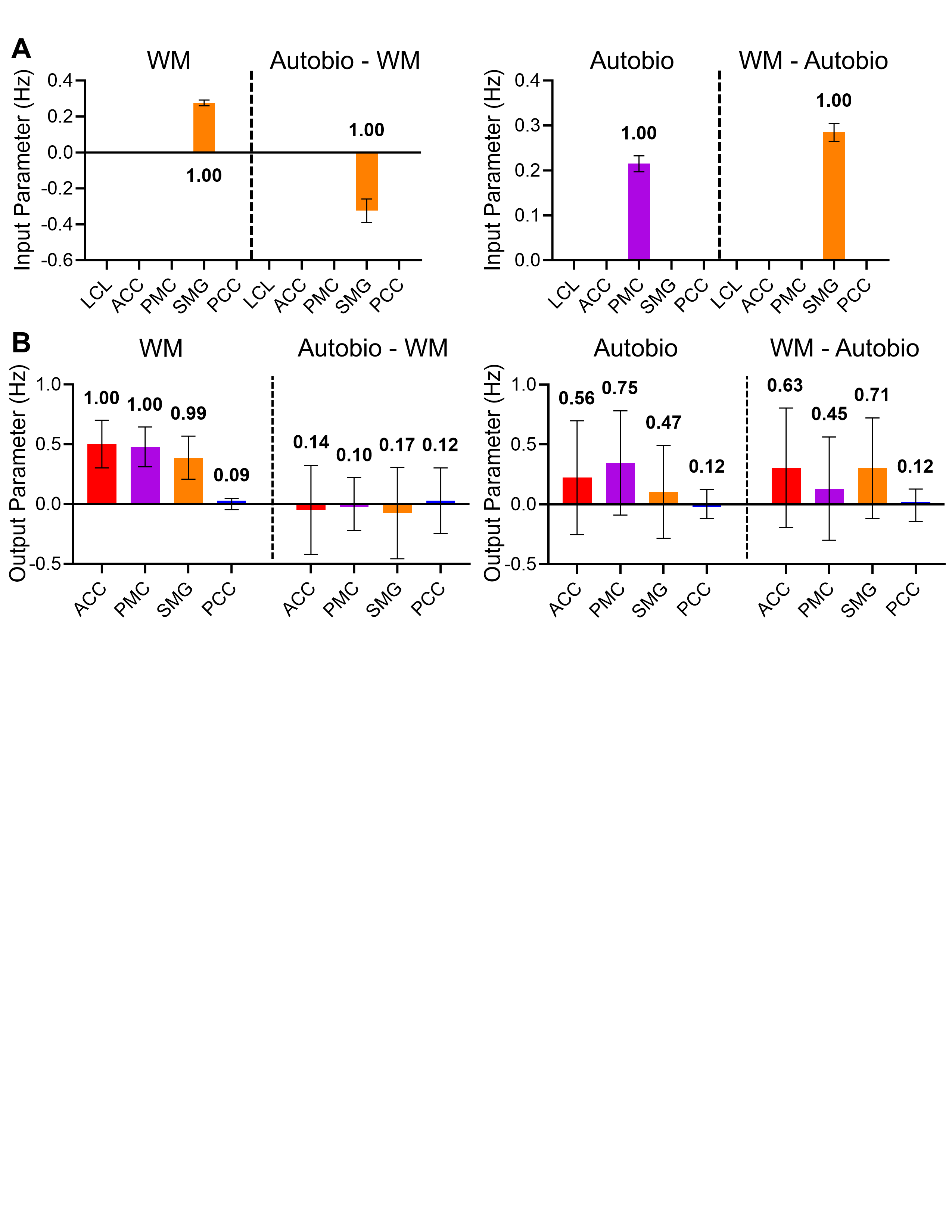

Supplement: S10 Fig — (A) Left: Estimated DCM input parameters during WM and differences with Autobio. Right: Estimated DCM input parameters during Autobio and differences with WM. (B) Left: Estimated DCM output parameters during WM and differences with Autobio. Right: Estimated DCM output parameters during Autobio and differences with WM. 95% confidence intervals and posterior probabilities signifying evidence strength (>0.5 = “weak”, >0.75 = “positive”, >0.95 = “strong”, >0.99 = “very strong”) accompany bars. The data and code used to produce this figure can be found in https://osf.io/akps6. (TIF) [file pbio.3003843.s012.TIF]

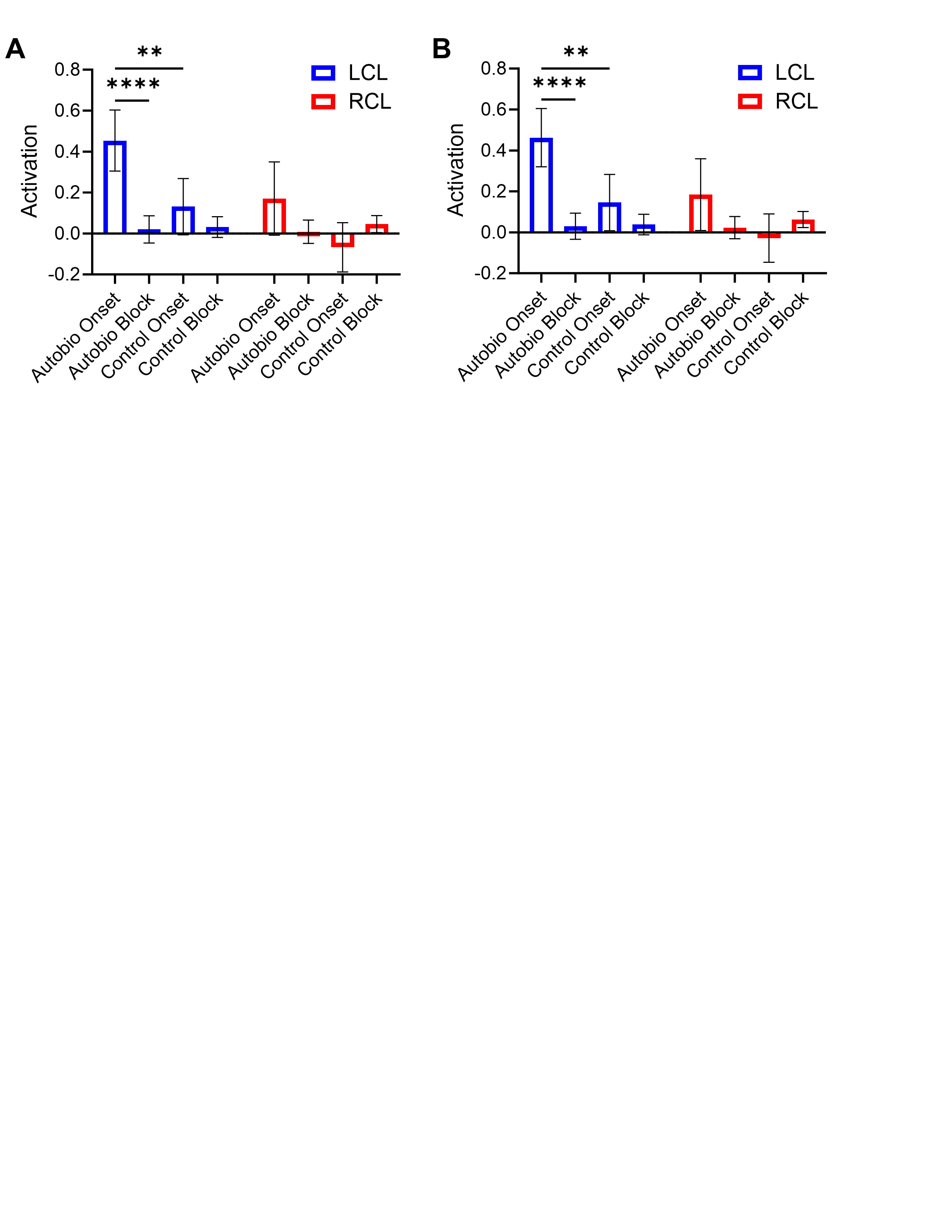

Supplement: S11 Fig — (A) LCL and RCL activation during autobiographical memory scans, reproduced from Fig 2C. (B) LCL and RCL activation during autobiographical memory scans following preprocessing which included removal of the initial 10 volumes. Bilateral claustrum results are qualitatively similar (2-way ANOVA main effect of condition: F (2.149, 146.2) = 13.69, p < 0.0001; main effect of hemisphere: F (1, 68) = 8.080, p = 0.0059; condition x hemisphere interaction: F (3, 204) = 3.559, p = 0.0152; post hoc LCL Autobio onset versus Autobio block: p < 0.0001; post hoc LCL Autobio onset vs. control onset: p = 0.0056). Bar graphs display means with 95% confidence intervals. The data underlying this figure can be found in S2 Data. (TIF) [file pbio.3003843.s013.TIF]

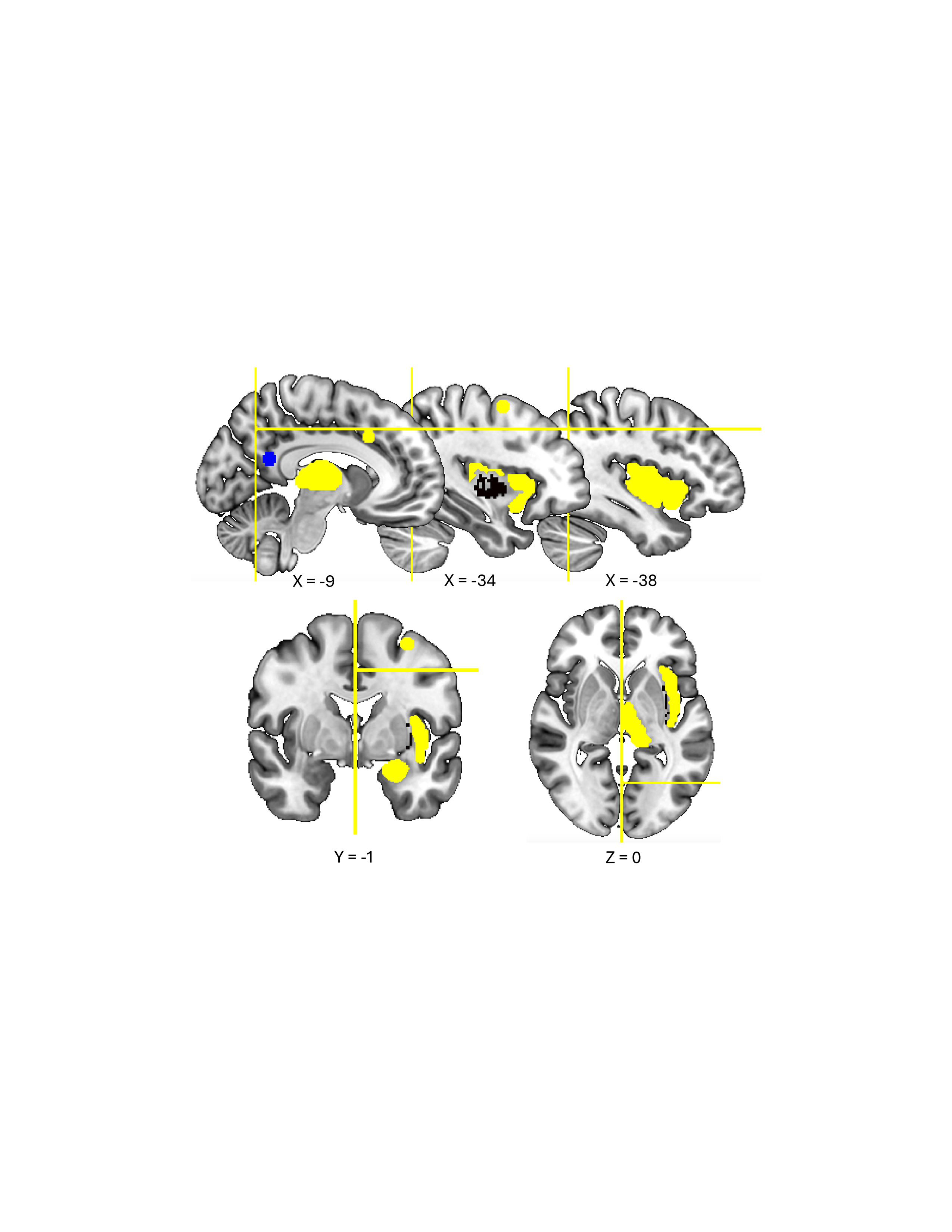

Supplement: S12 Fig — Representative structural connectivity analysis exclusion mask (yellow) setup for the connection between LCL (black, top middle) and PCC (blue, top left). Analyses used combinations of unanalyzed target ROIs, nearby notable ROIs, and additional planes for specificity. LCL-PCC analyses used ROI exclusion masks for ACC (top left), PMC (top middle, bottom left), SMG, and Hipp, as well as masks for thalamus, insular cortex, and amygdala. Exclusion planes for LCL-PCC included a mid-sagittal plane (x = 0), an axial plane at z = 42, and a coronal plane at y = −68. Images displayed in radiological orientation. (TIF) [file pbio.3003843.s014.TIF]
