# Supplementary material for: The human claustrum supports cognitive networks for externally and internally driven task demands
Source: PLoS Biol. 2026 Jun 26;24(6):e3003843. doi: 10.1371/journal.pbio.3003843 (PMC13308805; doi:10.1371/journal.pbio.3003843)
Supplement: S1 Table — Variance Inflation Factors (VIFs) reveal high likelihood of misattribution when modeling each task component (alert, encoding, retention, test, response) of working memory and control trials. Common “rules of thumb” regard VIFs exceeding 5 or 10 as suggesting high levels of multicollinearity that can severely impact model stability [84]. (PDF) [file pbio.3003843.s015.pdf]

	Working Memory Trials				
Regressor	Alert	Encoding	Retention	Test	Response
VIF	149.18	640.43	675.06	443.29	82.17
	Control Trials				
Regressors	Alert	Encoding	Retention	Test	Response
VIF	257.74	1215.50	1345.83	824.19	138.07

S1 Table. High likelihood of misattribution in working memory GLMs with individual task component regressors

Variance Inflation Factors (VIFs) reveal high likelihood of misattribution when modeling each task component (alert, encoding, retention, test, response) of working memory and control trials. Common “rules of thumb” regard VIFs exceeding 5 or 10 as suggesting high levels of multicollinearity that can severely impact model stability (O’Brien, 2007).
