# Supplementary material for: The human claustrum supports cognitive networks for externally and internally driven task demands
Source: PLoS Biol. 2026 Jun 26;24(6):e3003843. doi: 10.1371/journal.pbio.3003843 (PMC13308805; doi:10.1371/journal.pbio.3003843)
Supplement: S2 Table — Spearman r tests (average framewise displacement [FD] values were not normally distributed) find no significant correlations between individual participant LCL or RCL BOLD signal activation parameters and average motion during task scans. Working memory correlations used the working memory condition vs. implicit baseline contrast, and autobiographical memory correlations used the autobiographical memory onset condition vs. implicit baseline contrast. Implicit baseline in both models encompassed inter-trial intervals (ITIs) and volumes obtained prior to and following task runs. Only RCL-AVG FD during PIOP1 working memory yielded an uncorrected p-value less than 0.05 (r = −0.1578; p < 0.0264), but the inverse correlation revealed RCL signal decreased with greater motion, meaning motion-induced noise potentially biased observations away from significant condition effects. (PDF) [file pbio.3003843.s016.pdf]

Task & Dataset	LCL-AVG FD	RCL-AVG FD
Working Memory (PIOP1)<br>WM vs. baseline	*r* = -0.01568<br>*p* = 0.8265<br>*p*-FDR = 0.8449	*r* = -0.1578<br>*p* = 0.0264*<br>*p*-FDR = 0.1584
Working Memory (PIOP2)<br>WM vs. baseline	*r* = -0.01321<br>*p* = 0.8449<br>*p*-FDR = 0.8449	*r* = 0.02156<br>*p* = 0.7493<br>*p*-FDR = 0.8449
Autobiographical Memory<br>Autobio onset vs. baseline	*r* = 0.05602<br>*p* = 0.7492<br>*p*-FDR = 0.8449	*r* = 0.2793<br>*p* = 0.1042<br>*p*-FDR = 0.3126

S2 Table. No significant positive correlations between subject motion and claustrum BOLD signal change

Spearman *r* tests (average framewise displacement [FD] values were not normally distributed) find no significant correlations between individual participant LCL or RCL BOLD signal activation parameters and average motion during task scans. Working memory correlations used the working memory condition vs. implicit baseline contrast, and autobiographical memory correlations used the autobiographical memory onset condition vs. implicit baseline contrast. Implicit baseline in both models encompassed inter-trial intervals (ITIs) and volumes obtained prior to and following task runs. Only RCL-AVG FD during PIOP1 working memory yielded an uncorrected *p*-value less than 0.05 ($r = -0.1578$; $p < 0.0264$), but the inverse correlation revealed RCL signal decreased with greater motion, meaning motion-induced noise potentially biased observations away from significant condition effects.
