# Supplementary material for: The human claustrum supports cognitive networks for externally and internally driven task demands
Source: PLoS Biol. 2026 Jun 26;24(6):e3003843. doi: 10.1371/journal.pbio.3003843 (PMC13308805; doi:10.1371/journal.pbio.3003843)
Supplement: S3 Table — Working memory trials with responses were classified as correct or incorrect. If no response was made in the 1 s response phase, the trial was classified as a timeout. Control trials were only classified depending on if a response was made (“hit”) or not (“miss”) regardless of button press accuracy. Accuracy (defined as percent correct) was compared between working memory “different” and “match” trials. Because accuracy percentages were not normally distributed, Wilcoxon matched-pairs signed rank tests were performed to compare accuracies between conditions. “Different” and “match” accuracies were not significantly different in either dataset (PIOP1: p = 0.0797; PIOP2: p = 0.1815). (PDF) [file pbio.3003843.s017.pdf]

	All Working Memory			All Control		Working Memory: Different			Working Memory: Match		
Dataset	Correct	Incorrect	Timeout	Hit	Miss	Correct	Incorrect	Timeout	Correct	Incorrect	Timeout
PIOP1	53%	21%	26%	49%	51%	55%	18%	28%	51%	25%	24%
PIOP2	51%	47%	2%	73%	27%	52%	46%	2%	50%	48%	2%

S3 Table. Working memory task accuracy by trial type

Working memory trials with responses were classified as correct or incorrect. If no response was made in the 1s response phase, the trial was classified as a timeout. Control trials were only classified depending on if a response was made (“hit”) or not (“miss”) regardless of button press accuracy. Accuracy (defined as percent correct) was compared between working memory “different” and “match” trials. Because accuracy percentages were not normally distributed, Wilcoxon matched-pairs signed rank tests were performed to compare accuracies between conditions. “Different” and “match” accuracies were not significantly different in either dataset (PIOP1: $p = 0.0797$; PIOP2: $p = 0.1815$).
