# Supplementary material for: The human claustrum supports cognitive networks for externally and internally driven task demands
Source: PLoS Biol. 2026 Jun 26;24(6):e3003843. doi: 10.1371/journal.pbio.3003843 (PMC13308805; doi:10.1371/journal.pbio.3003843)
Supplement: S4 Table — Mixed Effects Analysis did not detect a significant effect of condition (different vs. match) or accuracy (correct vs. incorrect) on mean reaction time in either PIOP1 or PIOP2. Only a condition x accuracy interaction was detected in PIOP1 (p = 0.0132), where incorrect responses were made faster than correct responses in “different” trials. (PDF) [file pbio.3003843.s018.pdf]

	All Working Memory		All Control	Working Memory: Different		Working Memory: Match	
Dataset	Correct	Incorrect	Hit	Correct	Incorrect	Correct	Incorrect
PIOP1	0.57s	0.58s	0.68s	0.58s	0.56s	0.57s	0.59s
PIOP2	0.45s	0.47s	0.88s	0.45s	0.48s	0.44s	0.46s

S4 Table. Working memory task mean reaction time by trial type

Mixed Effects Analysis did not detect a significant effect of condition (different vs. match) or accuracy (correct vs. incorrect) on mean reaction time in either PIOP1 or PIOP2. Only a condition x accuracy interaction was detected in PIOP1 (p = 0.0132), where incorrect responses were made faster than correct responses in “different” trials.
