# Supplementary material for: The human claustrum supports cognitive networks for externally and internally driven task demands
Source: PLoS Biol. 2026 Jun 26;24(6):e3003843. doi: 10.1371/journal.pbio.3003843 (PMC13308805; doi:10.1371/journal.pbio.3003843)
Supplement: S5 Table — All VIFs using hybrid event-block format in autobiographical memory task GLMs are less than 2, reflecting no risk of model instability or misattribution. (PDF) [file pbio.3003843.s019.pdf]

Regressor	Autobio Onset	Autobio Block	Control Onset	Control Block
VIF	1.39	1.44	1.37	1.44

S5 Table. No risk of misattribution in autobiographical memory GLMs when using hybrid event-block format

All VIFs using hybrid event-block format in autobiographical memory task GLMs are less than 2, reflecting no risk of model instability or misattribution.
