# Supplementary material for: The human claustrum supports cognitive networks for externally and internally driven task demands
Source: PLoS Biol. 2026 Jun 26;24(6):e3003843. doi: 10.1371/journal.pbio.3003843 (PMC13308805; doi:10.1371/journal.pbio.3003843)
Supplement: S6 Table — Spearman r tests (average framewise displacement [FD] values were not normally distributed) find no significant correlations between individual participant LaINS BOLD signal activation and average motion during task scans. However, a significant positive correlation was found between LPulv BOLD signal activation and average motion during autobiographical memory scans (r = 0.5280, p-FDR = 0.0066), calling into question the validity of the observed task-induced BOLD increase in the region. Working memory correlations used the working memory condition vs. implicit baseline contrast, and autobiographical memory correlations used the autobiographical memory onset condition versus implicit baseline contrast. Implicit baseline in both models encompassed inter-trial intervals (ITIs) and volumes obtained prior to and following task runs. (PDF) [file pbio.3003843.s020.pdf]

Task & Dataset	LaINS-AVG FD	LPulv-AVG FD
Working Memory (PIOP1)<br>WM vs. baseline	*r* = -0.01522<br>*p* = 0.8314<br>*p*-FDR = 0.9953	*r* = -0.1326<br>*p* = 0.0626<br>*p*-FDR = 0.1252
Working Memory (PIOP2)<br>WM vs. baseline	*r* = 0.006146<br>*p* = 0.9274<br>*p*-FDR = 0.9953	*r* = 0.0003965<br>*p* = 0.9953<br>*p*-FDR = 0.9953
Autobiographical Memory<br>Autobio onset vs. baseline	*r* = 0.3258<br>*p* = 0.0562<br>*p*-FDR = 0.1252	*r* = 0.5280<br>*p* = 0.0011**<br>*p*-FDR = 0.0066**

S6 Table. Correlations between subject motion and LaINS and LPulv BOLD signal change

Spearman *r* tests (average framewise displacement [FD] values were not normally distributed) find no significant correlations between individual participant LaINS BOLD signal activation and average motion during task scans. However, a significant positive correlation was found between LPulv BOLD signal activation and average motion during autobiographical memory scans (*r* = 0.5280, *p*-FDR = 0.0066), calling into question the validity of the observed task-induced BOLD increase in the region. Working memory correlations used the working memory condition vs. implicit baseline contrast, and autobiographical memory correlations used the autobiographical memory onset condition vs. implicit baseline contrast. Implicit baseline in both models encompassed inter-trial intervals (ITIs) and volumes obtained prior to and following task runs.
