# Supplementary material for: The human claustrum supports cognitive networks for externally and internally driven task demands
Source: PLoS Biol. 2026 Jun 26;24(6):e3003843. doi: 10.1371/journal.pbio.3003843 (PMC13308805; doi:10.1371/journal.pbio.3003843)
Supplement: S7 Table — For each seed region (LCL, LaINS, LPulv) structural connectivity with that region was compared between all pairs of network representative regions in the combined AOMIC PIOP1&2 dataset. For example, the first row compares structural connectivity strength between LCL-PCC and LCL-Hipp connections, with LCL-PCC exhibiting significantly greater structural connectivity. Rows are arranged so the two strongest connections for any seed are compared in that seed’s bottom row. All p-values are adjusted using Bonferroni correction within each seed analysis. (PDF) [file pbio.3003843.s021.pdf]

Seed Region	Connections Compared	Standardized Test Statistic (*z*)	Adjusted Significance (*p*)
LCL	PCC > Hipp	6.83	<0.001
	ACC > Hipp	8.751	<0.001
	SMG > Hipp	21.713	<0.001
	PMC > Hipp	28.281	<0.001
	ACC > PCC	1.92	0.548
	SMG > PCC	14.882	<0.001
	PMC > PCC	21.451	<0.001
	SMG > ACC	12.962	<0.001
	PMC > ACC	19.531	<0.001
	PMC > SMG	6.568	<0.001
LaINS	PCC > Hipp	9.438	<0.001
	SMG > Hipp	12.482	<0.001
	ACC > Hipp	14.948	<0.001
	PMC > Hipp	25.488	<0.001
	SMG > PCC	3.044	0.023
	ACC > PCC	5.51	<0.001
	PMC > PCC	16.05	<0.001
	ACC > SMG	2.466	0.137
	PMC > SMG	13.006	<0.001
	PMC > ACC	10.54	<0.001
LPulv	SMG > ACC	3.11	0.019
	PCC > ACC	4.015	0.001
	PMC > ACC	11.009	<0.001
	Hipp > ACC	26.874	<0.001
	PCC > SMG	0.906	1.000
	PMC > SMG	7.899	<0.001
	Hipp > SMG	23.764	<0.001
	PMC > PCC	6.994	<0.001
	Hipp > PCC	22.858	<0.001
	Hipp > PMC	15.864	<0.001

S7 Table. Within-seed region structural connectivity post hoc comparison *p*-values

For each seed region (LCL, LaINS, LPulv) structural connectivity with that region was compared between all pairs of network representative regions in the combined AOMIC PIOP1&2 dataset. For example, the first row compares structural connectivity strength between LCL-PCC and LCL-Hipp connections, with LCL-PCC exhibiting significantly greater structural connectivity. Rows are arranged so the two strongest connections for any seed are compared in that seed's bottom row. All *p*-values are adjusted using Bonferroni correction within each seed analysis.
