# Supplementary material for: The human claustrum supports cognitive networks for externally and internally driven task demands
Source: PLoS Biol. 2026 Jun 26;24(6):e3003843. doi: 10.1371/journal.pbio.3003843 (PMC13308805; doi:10.1371/journal.pbio.3003843)
Supplement: S8 Table — For each network region, structural connectivity with that region was compared between all pairs of seed regions in the combined AOMIC PIOP1&2 dataset. For example, the first row compares structural connectivity strength between LPulv-ACC and LaINS-ACC connections, with LPulv-ACC exhibiting significantly stronger structural connectivity. Rows are arranged so the two strongest connections for any network region are compared in that region’s bottom row. All p-values are adjusted using Bonferroni correction within each region analysis. Because comparisons include 5 separate ANOVAs, significance is set at p < 0.05/5, or p < 0.01. (PDF) [file pbio.3003843.s022.pdf]

Network Region	Connections Compared	Standardized Test Statistic (*z*)	Adjusted Significance (*p*)
ACC (salience network)	LPulv > LaINS	15.768	<0.001
	LCL > LaINS	20.978	<0.001
	LCL > LPulv	5.21	<0.001
PMC (fronto-parietal network)	LPulv > LaINS	10.144	<0.001
	LCL > LaINS	24.118	<0.001
	LCL > LPulv	13.974	<0.001
SMG (dorsal attention network)	LPulv > LaINS	13.422	<0.001
	LCL > LaINS	26.43	<0.001
	LCL > LPulv	13.008	<0.001
PCC (default mode network)	LCL > LaINS	18.25	<0.001
	LPulv > LaINS	20.202	<0.001
	LPulv > LCL	1.949	0.154
Hipp	LCL > LaINS	14.04	<0.001
	LPulv > LaINS	28.76	<0.001
	LPulv > LCL	14.716	<0.001

S8 Table. Between-seed ROI structural connectivity post hoc comparison *p*-values

For each network region, structural connectivity with that region was compared between all pairs of seed regions in the combined AOMIC PIOP1&2 dataset. For example, the first row compares structural connectivity strength between LPulv-ACC and LaINS-ACC connections, with LPulv-ACC exhibiting significantly stronger structural connectivity. Rows are arranged so the two strongest connections for any network region are compared in that region's bottom row. All *p*-values are adjusted using Bonferroni correction within each region analysis. Because comparisons include 5 separate ANOVAs, significance is set at $p < 0.05/5$, or $p < 0.01$.
