# Supplementary material for: The human claustrum supports cognitive networks for externally and internally driven task demands
Source: PLoS Biol. 2026 Jun 26;24(6):e3003843. doi: 10.1371/journal.pbio.3003843 (PMC13308805; doi:10.1371/journal.pbio.3003843)
Supplement: S9 Table — Left claustrum exhibited statistically significant ROI-ROI functional connectivity at rest in the combined AOMIC PIOP1&2 dataset with all selected network representative ROIs and with all identified cognitive control networks. Significance of ROI-ROI resting state functional connectivity was calculated using paired-sample t-tests in MATLAB. (PDF) [file pbio.3003843.s023.pdf]

**Target Regions**	z	*p*
ACC (salience)	0.3437	2.35E-112
PMC (fronto-parietal)	0.1915	1.83E-52
SMG (dorsal attention)	0.2074	4.03E-57
PCC (default mode)	0.1337	1.46E-34
**Target Networks**		
Salience	0.4206	3.03E-124
Left Fronto-Parietal	0.3402	1.36E-107
Right Fronto-Parietal	0.2290	5.52E-61
Dorsal Attention	0.3474	3.22E-107
Default Mode	0.2345	3.89E-71

S9 Table. Left claustrum resting state functional connectivity with networks and network representative regions

Left claustrum exhibited statistically significant ROI-ROI functional connectivity at rest in the combined AOMIC PIOP1&2 dataset with all selected network representative ROIs and with all identified cognitive control networks. Significance of ROI-ROI resting state functional connectivity was calculated using paired-sample t-tests in MATLAB.
