# Supplementary material for: The human claustrum supports cognitive networks for externally and internally driven task demands
Source: PLoS Biol. 2026 Jun 26;24(6):e3003843. doi: 10.1371/journal.pbio.3003843 (PMC13308805; doi:10.1371/journal.pbio.3003843)
Supplement: S10 Table — Leave-one-out cross-validation was performed to assess model effectiveness in predicting task state (working memory using combined PIOP1&2 datasets vs. autobiographical memory) and seed region. Pearson’s correlation coefficients, uncorrected p-values, and p values after Benjamini–Hochberg False Discovery Rate correction accounting for all statistical tests in the table are listed. Note the similarity in results when using “input” parameters across all seed regions. Seed region output parameters were statistically significant in distinguishing LCL vs. LaINS during working memory, with a trend toward significance in the smaller autobiographical memory dataset. (PDF) [file pbio.3003843.s024.pdf]

Task-State Prediction: Working Memory vs. Autobiographical Memory					
	Parameters	df	r	p	p-FDR
LCL	Input	453	0.40	<0.001***	<0.001***
	Output	453	-0.14	0.999	0.999
LaINS	Input	453	0.40	<0.001***	<0.001***
	Output	453	0.02	0.344	0.574
LPulv	Input	453	0.39	<0.001***	<0.001***
	Output	453	-0.16	0.999	0.999
**Seed Region Prediction**					
LCL vs. LaINS	WM Output	838	0.17	<0.001***	<0.001***
	Autobio Output	68	0.20	0.045*	0.089
LCL vs. LPulv	WM Output	838	-0.01	0.601	0.858
	Autobio Output	68	-0.27	0.989	0.999

S10 Table. Dynamic causal modeling cross-validation

Leave-one out cross-validation was performed to assess model effectiveness in predicting task state (working memory using combined PIOP1&2 datasets vs. autobiographical memory) and seed region. Pearson's correlation coefficients, uncorrected *p*-values, and *p* values after Benjamini-Hochberg False Discovery Rate correction accounting for all statistical tests in the table are listed. Note the similarity in results when using "input" parameters across all seed regions. Seed region output parameters were statistically significant in distinguishing LCL vs. LaINS during working memory, with a trend toward significance in the smaller autobiographical memory dataset.
