# Supplementary material for: The human claustrum supports cognitive networks for externally and internally driven task demands
Source: PLoS Biol. 2026 Jun 26;24(6):e3003843. doi: 10.1371/journal.pbio.3003843 (PMC13308805; doi:10.1371/journal.pbio.3003843)
Supplement: S11 Table — Probabilities assigned to sources of experimental input to seed regions during working and autobiographical memory tasks. In all models the seed region was used as the fifth possible source of input, modeling all other possible sources of experimental input. Note that input source probabilities are identical across seed regions. (PDF) [file pbio.3003843.s025.pdf]

Seed Region	Task	ACC	PMC	SMG	PCC	Other
LCL	Working Memory	0.00	0.00	1.00	0.00	0.00
	Autobiographical Memory	0.00	1.00	0.00	0.00	0.00
LaINS	Working Memory	0.00	0.00	1.00	0.00	0.00
	Autobiographical Memory	0.00	1.00	0.00	0.00	0.00
LPulv	Working Memory	0.00	0.00	1.00	0.00	0.00
	Autobiographical Memory	0.00	1.00	0.00	0.00	0.00

S11 Table. Input Bayesian model comparison probabilities

Probabilities assigned to sources of experimental input to seed regions during working and autobiographical memory tasks. In all models the seed region was used as the fifth possible source of input, modeling all other possible sources of experimental input. Note that input source probabilities are identical across seed regions.
