# Supplementary material for: The human claustrum supports cognitive networks for externally and internally driven task demands
Source: PLoS Biol. 2026 Jun 26;24(6):e3003843. doi: 10.1371/journal.pbio.3003843 (PMC13308805; doi:10.1371/journal.pbio.3003843)
Supplement: S12 Table — Probabilities assigned to models with different combinations of task-modulation of network representative region outputs. Values reveal which model structure is favored, but they do not provide insight into the parameters (e.g., excitatory or inhibitory) describing the models. (PDF) [file pbio.3003843.s026.pdf]

ROI	Task	1	2	3	4	5	6	7	8	9	10	11	12	13	14	15	16
LCL	WM	0.09	0.90	0.00	0.01	0.00	0.00	0.00	0.00	0.00	0.00	0.00	0.00	0.00	0.00	0.00	0.00
	Autobio - WM	0.00	0.00	0.00	0.01	0.00	0.02	0.01	0.09	0.00	0.01	0.01	0.07	0.02	0.11	0.08	0.56
	Autobio	0.02	0.18	0.03	0.20	0.01	0.06	0.01	0.07	0.02	0.14	0.02	0.15	0.01	0.05	0.01	0.05
	WM - Autobio	0.02	0.18	0.01	0.07	0.03	0.22	0.01	0.09	0.01	0.10	0.01	0.04	0.02	0.13	0.01	0.05
LaINS	WM	0.08	0.92	0.00	0.00	0.00	0.00	0.00	0.00	0.00	0.00	0.00	0.00	0.00	0.00	0.00	0.00
	Autobio - WM	0.00	0.00	0.00	0.00	0.01	0.01	0.03	0.03	0.01	0.01	0.03	0.04	0.10	0.11	0.29	0.32
	Autobio	0.03	0.12	0.05	0.22	0.03	0.11	0.04	0.19	0.01	0.03	0.01	0.06	0.01	0.03	0.01	0.05
	WM - Autobio	0.04	0.16	0.01	0.03	0.03	0.10	0.01	0.02	0.06	0.24	0.01	0.05	0.04	0.15	0.01	0.03
LPulv	WM	0.04	0.26	0.05	0.34	0.01	0.06	0.01	0.08	0.01	0.04	0.01	0.06	0.00	0.01	0.00	0.02
	Autobio - WM	0.00	0.00	0.00	0.01	0.00	0.01	0.01	0.08	0.00	0.01	0.01	0.08	0.01	0.07	0.07	0.63
	Autobio	0.01	0.08	0.02	0.14	0.02	0.09	0.03	0.14	0.01	0.07	0.02	0.12	0.01	0.08	0.02	0.13
	WM - Autobio	0.02	0.10	0.03	0.18	0.01	0.07	0.02	0.13	0.01	0.08	0.03	0.14	0.01	0.05	0.02	0.10

S12 Table. Output Bayesian model comparison probabilities

Probabilities assigned to models with different combinations of task-modulation of network representative region outputs. Values reveal which model structure is favored, but they do not provide insight into the parameters (e.g., excitatory or inhibitory) describing the models.
